# Supplementary material for: Comparing lagged impacts of mobility changes and environmental factors on COVID-19 waves in rural and urban India: A Bayesian spatiotemporal modelling study
Source: PLOS Glob Public Health. 2025 Apr 30;5(4):e0003431. doi: 10.1371/journal.pgph.0003431 (PMC12043145; doi:10.1371/journal.pgph.0003431)
Supplement: S1 Fig — Areas shaded in grey are areas for which no data is available. S2 Fig. Five periods for travel network modularity analysis (A): 1) Pre-pandemic period (15 weeks) from November 10, 2019 to February 22, 2020; 2) First lockdown (6 weeks), from March 22 to May 2, 2020, that included strict travel restrictions, stay-at home orders and closure of many businesses; 3) Pre-second lockdown period (8 weeks) from January 31 to March 27, 2021; 4) Second lockdown (6 weeks) for the Delta wave, from April 18 to May 29, 2021; 5) post-second lockdown period (8 weeks), from November 7 to December 31, 2021, after travel restrictions for COVID-19 had been lifted in India. S3 Fig. Relative changes of outbound travel from districts across India during the pandemic compared with average pre-pandemic levels during the 12 weeks from November 10, 2019, to February 22, 2020. (A) Reductions of outbound flows under the first lockdown during the 6-week period from March 22 to May 2, 2020. (B) Changes in outflow during the 8-week period from January 31 to March 27, 2021, before the second lockdown. (C) Reductions of outflows during the 6-week second lockdown from April 18 to May 29, 2021. (D) Changes in outflow during the 8-week period from November 7 to December 31, 2021. Sub-division maps at administrative level I (state) and II (district) were obtained from the GADM version 3.6 (https://gadm.org/). Regions in which outflow data are not available are those represented in green. Areas shaded in grey are areas for which no data is available. S1 Table. Summary Statistics for data used for wave 1 and Delta wave spatiotemporal models S2 Table. Wave 2: Adequacy results for models with DLNMs and increasing complexity. S3 Table. Wave 2: Adequacy results for models (without DLNMs) using 2-week lag covariates with increasing complexity. S4 Table. Model hyperparameters using a range of prior distributions in best fit model 4.1 for Delta Wave. S4 Fig Relative intra-district mobility during the Delta wave in [file pgph.0003431.s001.docx]

**Supplementary Information**

**
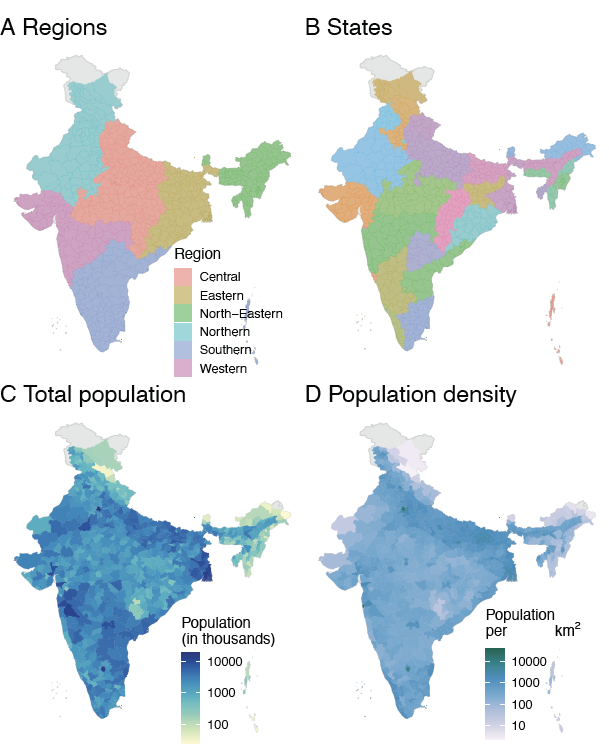
**

**S1 Fig**. Regions in India investigated by this study and the number and density of population at district level (administrative level II) in 2020. Areas shaded in grey are areas for which no data is available.


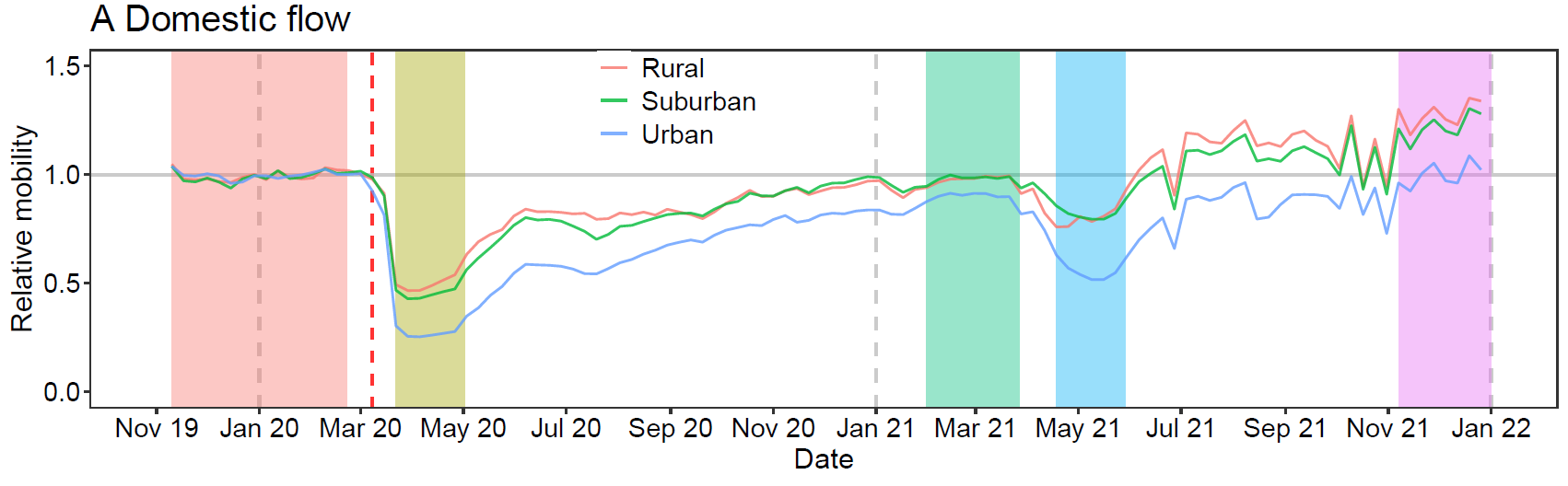


**S2 Fig**. Five periods for travel network modularity analysis (A): 1) Pre-pandemic period (15 weeks) from November 10, 2019 to February 22, 2020; 2) First lockdown (6 weeks), from March 22 to May 2, 2020, that included strict travel restrictions, stay-at home orders and closure of many businesses; 3) Pre-second lockdown period (8 weeks) from January 31 to March 27, 2021; 4) Second lockdown (6 weeks) for the Delta wave, from April 18 to May 29, 2021; 5) post-second lockdown period (8 weeks), from November 7 to December 31, 2021, after travel restrictions for COVID-19 had been lifted in India.


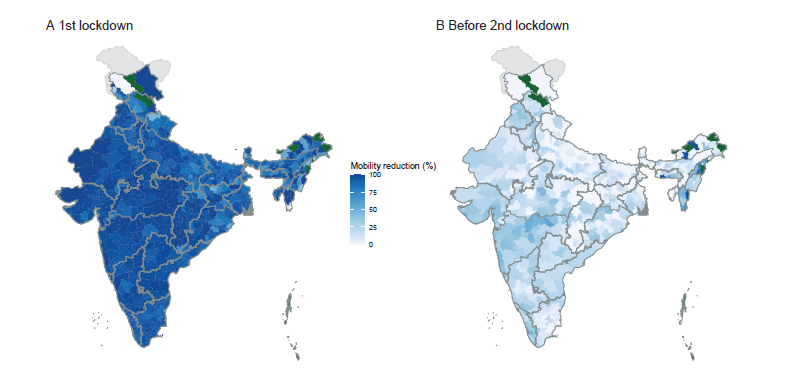


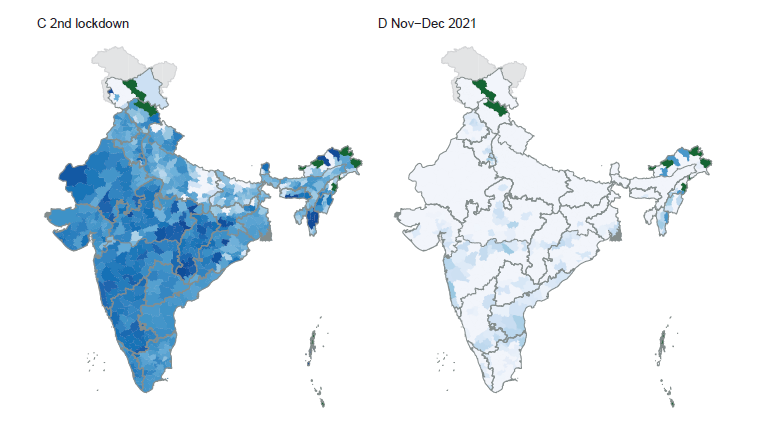
 **S3 Fig. Relative changes of outbound travel from districts across India during the pandemic compared with average pre-pandemic levels during the 12 weeks from November 10, 2019, to February 22, 2020.** (**A**) Reductions of outbound flows under the first lockdown during the 6-week period from March 22 to May 2, 2020. (**B**) Changes in outflow during the 8-week period from January 31 to March 27, 2021, before the second lockdown. (**C**) Reductions of outflows during the 6-week second lockdown from April 18 to May 29, 2021. (**D**) Changes in outflow during the 8-week period from November 7 to December 31, 2021. Sub-division maps at administrative level I (state) and II (district) were obtained from the GADM version 3.6 (<https://gadm.org/>). Regions in which outflow data are not available are those represented in green. Areas shaded in grey are areas for which no data is available.

| **S1 Table. Summary Statistics for data used for wave 1 and Delta wave spatiotemporal models** | | | | |
| --- | --- | --- | --- | --- |
|  | **Wave 1** | | **Delta Wave** | |
| **Variable** | **Mean** | **Standard Deviation** | **Mean** | **Standard Deviation** |
| R0 | 3.19 | 0.0260 | 4.40 | 0.272 |
| R | 1.08 | 0.360 | 1.27 | 0.747 |
| ln(R) | -1.13 | 0.280 | -1.38 | 0.557 |
| Cases (no.) | 671 | 1790 | 1796 | 5612 |
| Cases (rate) | 0.0422 | 0.119 | 0.116 | 0.386 |
| Cases (cumulative no.) | 7447 | 19719 | 15988 | 46227 |
| Cases (cumulative rate) | 0.461 | 1.44 | 0.985 | 3.26 |
| Mobility | 0.992 | 0.138 | 0.913 | 0.188 |
| Humidity | 20.4 | 6.58 | 18.0 | 6.45 |
| Temperature | 25.1 | 5.50 | 27.2 | 6.01 |
| Precipitation | 0.244 | 0.319 | 0.152 | 0.248 |
| UV Index | 75108 | 14949 | 103580 | 18888 |
| Stringency Index | 76.6 | 10.1 | 68.2 | 17.5 |
| Public Holidays | 1.2 | 1.66 | 1 | 1.33 |
| Population (total no.) | 2086588 | 1869856 | 2074997 | 1870190 |
| Population density | 853 | 2692 | 853 | 2685 |

**Delta Wave**

**S2 Table. Wave 2: Adequacy results for models with DLNMs and increasing complexity.**

| Model | COVID-19 transmission risk Rt/R0 | Whole country | | Urban | | Suburban | | Rural | |
| --- | --- | --- | --- | --- | --- | --- | --- | --- | --- |
|  |  | DIC | LS | DIC | LS | DIC | LS | DIC | LS |
| Base model | Spatiotemporal random effects + D | 7213 | 0.324 | 2746 | 0.297 | 2917 | 0.283 | 1192 | 0.45 |
| 1.1 | Base model + M | 7013 | 0.316 | 2563 | 0.277 | 2868 | 0.279 | 1193 | 0.45 |
| 1.2 | Base model + T | 7113 | 0.32 | 2618 | 0.283 | 2907 | 0.282 | 1197 | 0.451 |
| 1.3 | Base model + P | 7180 | 0.323 | 2671 | 0.288 | 2916 | 0.283 | 1196 | 0.451 |
| 1.4 | Base model + S | 6842 | 0.307 | 2539 | 0.274 | 2806 | 0.272 | 1161* | 0.437 |
| 2.1 | Base model + M + T | 6964 | 0.313 | 2485 | 0.269 | 2868 | 0.278 | 1197 | 0.452 |
| 2.2 | Base model + M + P | 6976 | 0.314 | 2502 | 0.27 | 2870 | 0.279 | 1197 | 0.451 |
| 2.3 | Base model + M + S | 6715 | 0.302 | 2381 | 0.257 | 2783 | 0.27 | 1170 | 0.441 |
| 2.4 | Base model + M + H | 7013 | 0.316 | 2563 | 0.277 | 2868 | 0.279 | 1194 | 0.45 |
| 3.1 | Base model + M + T + P | 6936 | 0.312 | 2465 | 0.267 | 2869 | 0.278 | 1206 | 0.455 |
| 3.2 | Base model + M + T + S | 6652 | 0.299 | 2302 | 0.248 | 2779* | 0.27 | 1170 | 0.442 |
| 3.3 | Base model + M + T + H | 6964 | 0.313 | 2485 | 0.269 | 2868 | 0.279 | 1198 | 0.452 |
| 3.4 | Base model + M + P + S | 6685 | 0.3 | 2320 | 0.25 | 2788 | 0.27 | 1174 | 0.443 |
| 3.5 | Base model + M + P + H | 6976 | 0.314 | 2502 | 0.27 | 2870 | 0.279 | 1197 | 0.452 |
| 3.6 | Base model + M + S + H | 6716 | 0.302 | 2381 | 0.257 | 2784 | 0.27 | 1170 | 0.441 |
| 4.1 | Base model + M + T + P + S | 6633* | 0.298 | 2285* | 0.247 | 2785 | 0.27 | 1178 | 0.445 |
| 4.2 | Base model + M + T + P + H | 6933 | 0.312 | 2466 | 0.267 | 2869 | 0.278 | 1206 | 0.455 |
| 4.3 | Base model + M + T + S + H | 6650 | 0.299 | 2303 | 0.248 | 2780 | 0.27 | 1171 | 0.442 |
| 4.4 | Base model + M + P + S + H | 6685 | 0.3 | 2319 | 0.25 | 2788 | 0.27 | 1175 | 0.443 |

DIC: deviance information criterion; LS: logarithmic score; D: cumulative cases per 1000 people since the first week in the modelling; M: mobility with Distributed Lag Nonlinear Model (DLNM); T: temperature with DLNM; P: precipitation with DLNM; S: stringency index of intervention policy with DLNM; H: public holidays. *DIC of the best fitting model for each region.

**S3 Table. Wave 2: Adequacy results for models (without DLNMs) using 2-week lag covariates with increasing complexity.**

| Model | COVID-19 transmission risk Rt/R0 | Whole country | | Urban | | Suburban | | Rural | |
| --- | --- | --- | --- | --- | --- | --- | --- | --- | --- |
|  |  | DIC | LS | DIC | LS | DIC | LS | DIC | LS |
| Base model | Spatiotemporal random effects + D | 7222 | 0.325 | 2763 | 0.298 | 2918 | 0.283 | 1194 | 0.45 |
| 1.1 | Base model + M | 6935 | 0.312 | 2522 | 0.273 | 2839 | 0.276 | 1196 | 0.451 |
| 1.2 | Base model + T | 7007 | 0.315 | 2634 | 0.285 | 2869 | 0.279 | 1185 | 0.447 |
| 1.3 | Base model + UV | 7127 | 0.32 | 2646 | 0.286 | 2905 | 0.282 | 1195 | 0.451 |
| 1.4 | Base model + S | 7087 | 0.318 | 2714 | 0.293 | 2851 | 0.277 | 1185 | 0.446 |
| 2.1 | Base model + M + T | 6754 | 0.304 | 2447 | 0.266 | 2798 | 0.272 | 1187 | 0.448 |
| 2.2 | Base model + M + UV | 6841 | 0.308 | 2439 | 0.264 | 2822 | 0.274 | 1198 | 0.451 |
| 2.3 | Base model + M + S | 6861 | 0.309 | 2488 | 0.269 | 2798 | 0.272 | 1186 | 0.447 |
| 2.4 | Base model + M + H | 6936 | 0.312 | 2522 | 0.273 | 2839 | 0.276 | 1196 | 0.451 |
| 3.1 | Base model + M + T + UV | 6757 | 0.304 | 2430 | 0.263 | 2803 | 0.272 | 1192 | 0.449 |
| 3.2 | Base model + M + T + S | 6665 | 0.3 | 2395 | 0.26 | 2750* | 0.267 | 1181* | 0.445 |
| 3.3 | Base model + M + T + H | 6754 | 0.304 | 2448 | 0.265 | 2797 | 0.272 | 1188 | 0.448 |
| 3.4 | Base model + M + UV + S | 6737 | 0.303 | 2390 | 0.259 | 2769 | 0.269 | 1188 | 0.448 |
| 3.5 | Base model + M + UV + H | 6843 | 0.308 | 2439 | 0.264 | 2822 | 0.274 | 1198 | 0.452 |
| 3.6 | Base model + M + S + H | 6869 | 0.309 | 2488 | 0.269 | 2798 | 0.272 | 1187 | 0.447 |
| 4.1 | Base model + M + T + UV + S | 6658* | 0.299 | 2377* | 0.258 | 2750 | 0.267 | 1184 | 0.446 |
| 4.2 | Base model + M + T + UV + H | 6759 | 0.304 | 2429 | 0.263 | 2803 | 0.272 | 1192 | 0.45 |
| 4.3 | Base model + M + T + S + H | 6665 | 0.3 | 2396 | 0.26 | 2750 | 0.267 | 1181 | 0.445 |
| 4.4 | Base model + M + UV + S + H | 6737 | 0.303 | 2391 | 0.259 | 2769 | 0.269 | 1188 | 0.448 |

DIC: deviance information criterion; LS: logarithmic score; D: cumulative cases per 1000 people since the first week in the modelling; M: mobility; T: temperature; U: ultraviolet radiation; S: stringency index of intervention policy; H: public holidays. *DIC of the best fitting model for each region.

**Model Hyperparameters**

The penalized complexity (PC) prior was used with a range of parameter settings. The PC prior for the precision $\tau$ has density

$$\pi\left( \tau\right)=\frac{\lambda}{2}\tau^{-\frac{3}{2}}\exp\left( -\lambda\tau^{-\frac{1}{2}} \right), \tau>0$$

for $\lambda>0$ where

$$\lambda=-\frac{\ln\left( \alpha\right)}{u}$$

and $\left( u,\alpha\right)$ are the parameter to this prior. The interpretation of $\left( u,\alpha\right)$ is that

$$\Pr\left( \sigma>u \right)=\alpha, u>0, 0<\alpha<1,$$

Where the standard deviation is $\sigma=\frac{1}{\sqrt{\tau}}$.

| **S4 Table. Model hyperparameters using a range of prior distributions in best fit model 4.1 for Delta Wave** | | | | | | | |
| --- | --- | --- | --- | --- | --- | --- | --- |
| **Prior distributions** | **Model evaluations** | **Mean** | **SD** | **0.025 quantile** | **0.5 quantile** | **0.975 quantile** | **DIC** |
| $u=3\cdot SD$  $\alpha=0.01$ | base model | 12.726 | 0.174 | 12.390 | 12.724 | 13.075 | 6430.84 |
|  | rw1 | 53.736 | 16.287 | 27.297 | 51.884 | 91.015 |  |
|  | BYM2 | 42.286 | 3.392 | 35.493 | 42.347 | 48.793 |  |
|  |  |  |  |  |  |  |  |
| $u=2\cdot SD$  $\alpha=0.01$ | Precision for base model | 12.726 | 0.174 | 12.390 | 12.724 | 13.076 | 6429.89 |
|  | Precision for rw1 | 58.116 | 17.584 | 31.498 | 55.457 | 100.319 |  |
|  | Precision for BYM2 | 42.338 | 3.410 | 35.528 | 42.392 | 48.902 |  |
|  |  |  |  |  |  |  |  |
| $u=SD$  $\alpha=0.01$ | Precision for base model | 12.726 | 0.174 | 12.390 | 12.724 | 13.075 | 6431.23 |
|  | Precision for rw1 | 66.104 | 27.913 | 30.430 | 59.959 | 137.492 |  |
|  | Precision for BYM2 | 42.337 | 3.389 | 35.544 | 42.401 | 48.833 |  |
|  |  |  |  |  |  |  |  |
| $u=0.5$  $\alpha=0.001$ | Precision for base model | 12.726 | 0.174 | 12.389 | 12.724 | 13.075 | 6431.52 |
|  | Precision for rw1 | 75.040 | 36.819 | 33.238 | 5.602 | 171.807 |  |
|  | Precision for BYM2 | 42.488 | 3.389 | 35.710 | 42.547 | 48.997 |  |
|  |  |  |  |  |  |  |  |
| $u=0.5$  $\alpha=0.01$ | Precision for base model | 12.726 | 0.174 | 12.390 | 12.724 | 13.076 | 6429.52 |
|  | Precision for rw1 | 55.546 | 15.942 | 30.029 | 53.589 | 92.556 |  |
|  | Precision for BYM2 | 42.361 | 3.408 | 35.554 | 42.416 | 48.919 |  |
|  |  |  |  |  |  |  |  |
| $u=1$  $\alpha=0.01$ | Precision for base model | 12.726 | 0.174 | 12.390 | 12.724 | 13.075 | 6430.81 |
|  | Precision for rw1 | 53.802 | 16.268 | 27.399 | 51.950 | 91.045 |  |
|  | Precision for BYM2 | 42.292 | 3.390 | 35.505 | 42.352 | 48.798 |  |
|  |  |  |  |  |  |  |  |
| $u=1$  $\alpha=0.001$ | Precision for base model | 12.726 | 0.174 | 12.390 | 12.723 | 13.074 | 6431.12 |
|  | Precision for rw1 | 54.515 | 20.210 | 23.657 | 51.670 | 102.100 |  |
|  | Precision for BYM2 | 43.342 | 3.367 | 35.584 | 42.408 | 48.784 |  |
|  |  |  |  |  |  |  |  |
| $u=0.1$  $\alpha=0.01$ | Precision for base model | 12.723 | 0.174 | 12.388 | 12.721 | 13.073 | 6430.03 |
|  | Precision for rw1 | 64.513 | 18.827 | 37.287 | 61.286 | 110.867 |  |
|  | Precision for BYM2 | 43.016 | 3.386 | 36.212 | 43.087 | 49.483 |  |
|  |  |  |  |  |  |  |  |
| $u=0.1$  $\alpha=0.001$ | Precision for base model | 12.721 | 0.174 | 12.387 | 12.718 | 13.071 | 6430.26 |
|  | Precision for rw1 | 70.000 | 21.403 | 40.964 | 65.852 | 123.991 |  |
|  | Precision for BYM2 | 43.334 | 3.454 | 36.344 | 43.425 | 49.871 |  |

DIC: deviance information criterion. *DIC of the best fitting model (model 4.1). rw1 is the temporal random effect model, $r_{t}$; BYM2 is the spatial random effect model; SD=0.75 standard deviation of $\Delta R_{t}$ during the Delta Wave 1.


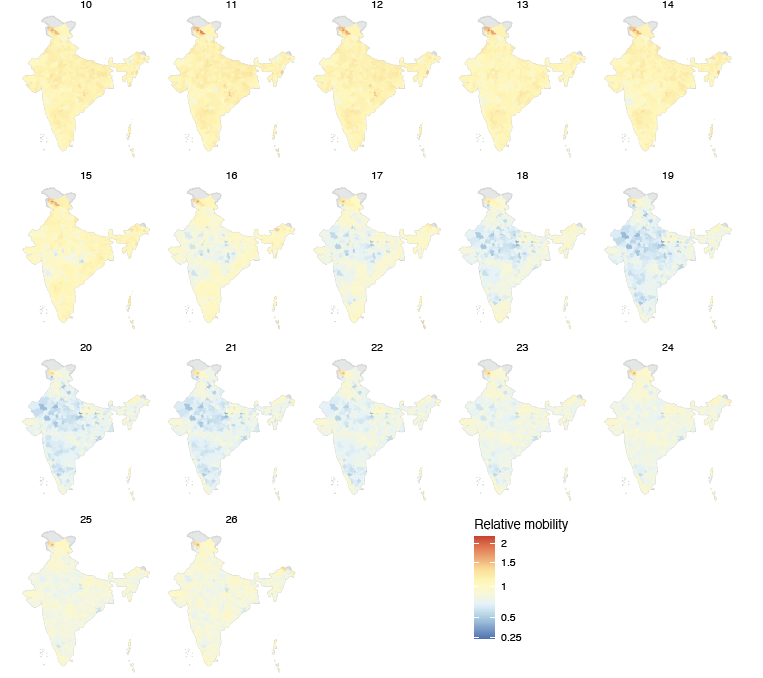


**S4 Fig**. Relative intra-district mobility during the Delta wave in India, standardised by pre-pandemic mean baseline levels of mobility for the first eight weeks of 2020 (December 29, 2019 – February 22, 2020) for each district. The weeks in 2021 investigated are numbered in maps. Areas shaded in grey are areas for which no data is available.


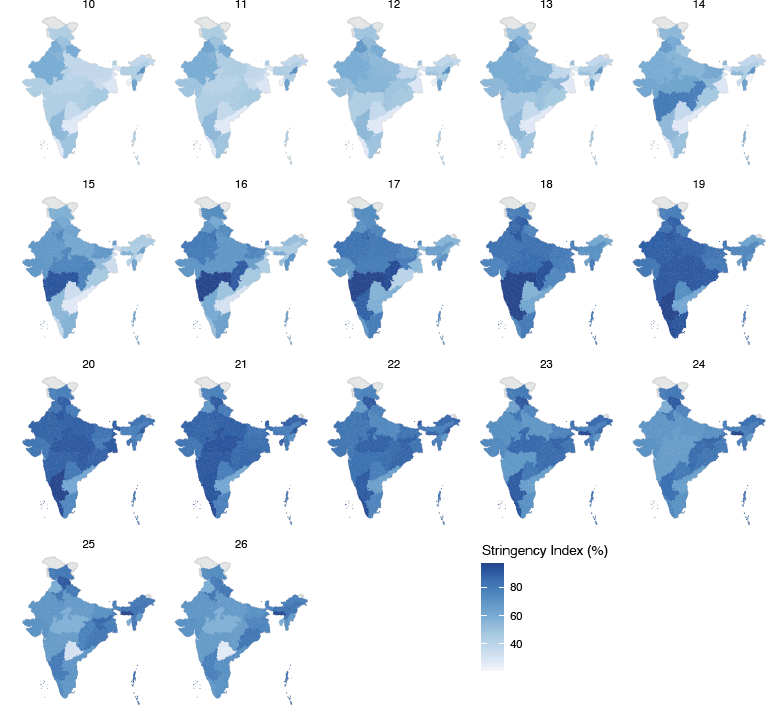


**S5 Fig**. Stringency Index of COVID-19 intervention policy implemented during the Delta wave in India. The weeks in 2021 investigated are numbered in maps. Areas shaded in grey are areas for which no data is available.

**
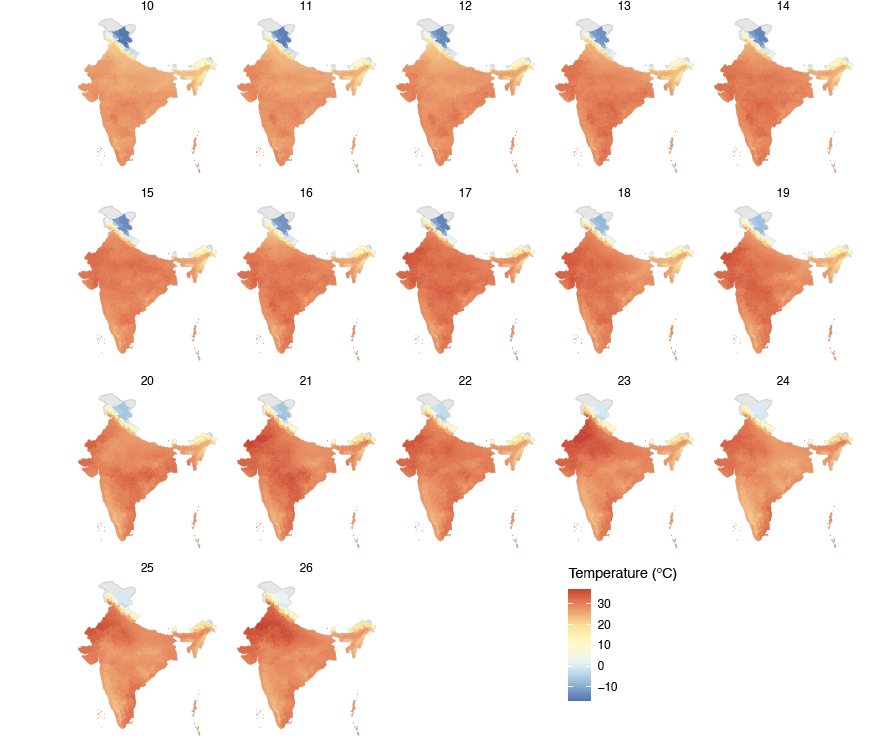
**

**S6 Fig**. Mean temperature at 2m above the surface during the Delta wave in India. The weeks in 2021 investigated are numbered in maps. Areas shaded in grey are areas for which no data is available.


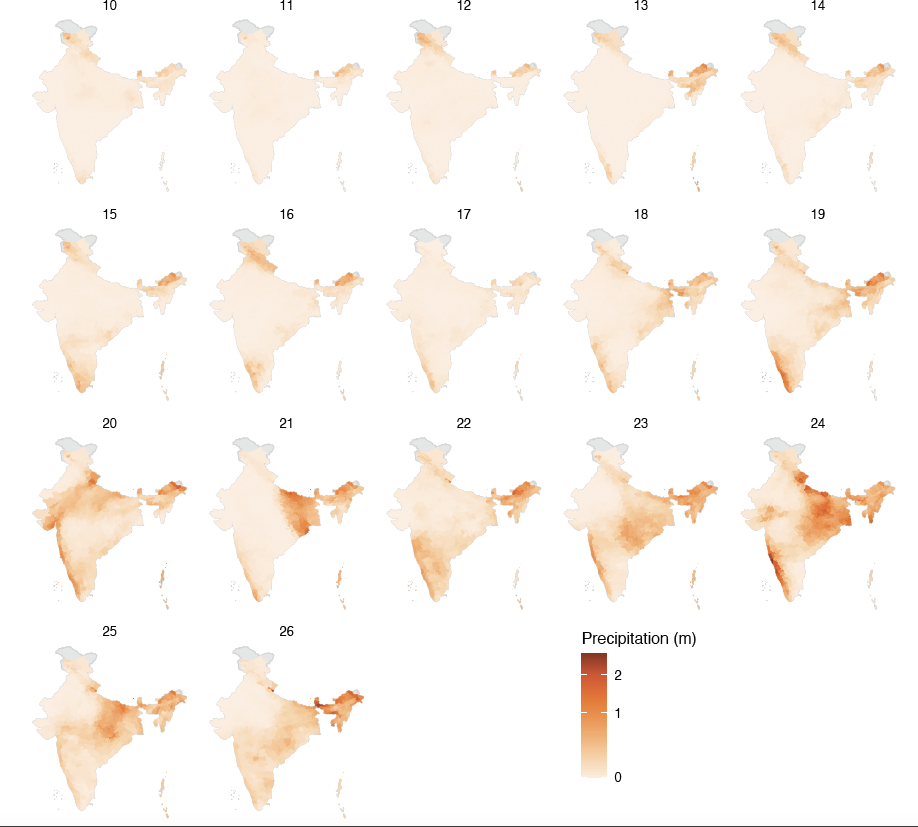


**S7 Fig**. Accumulated weekly precipitation (metres) during the Delta wave in India. The weeks in 2021 investigated are numbered in maps. Areas shaded in grey are areas for which no data is available.


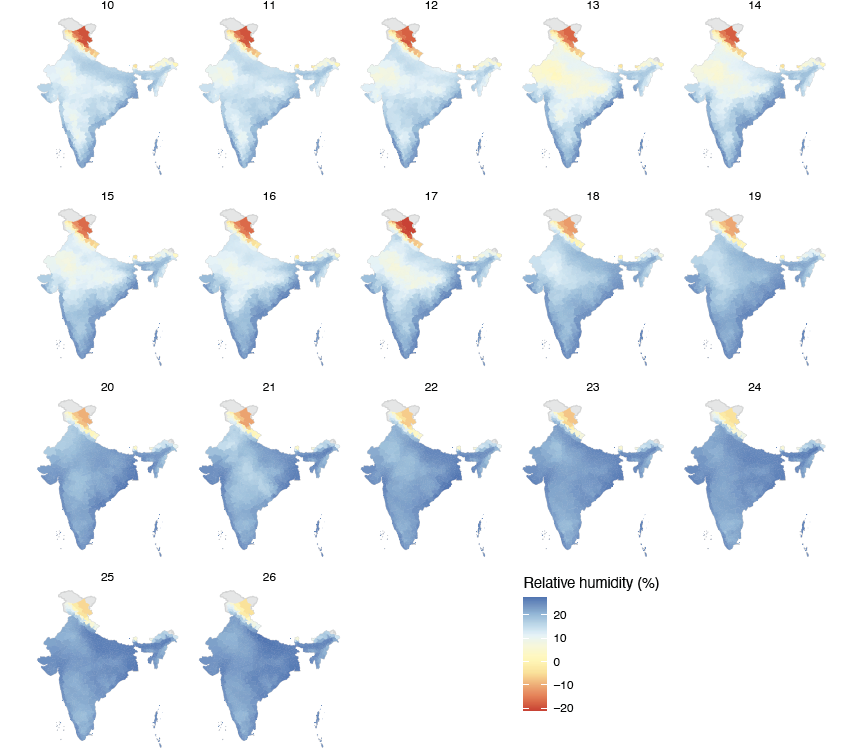


**S8 Fig**. Relative humidity during the Delta wave in India. The weeks in 2021 investigated are numbered in maps. Areas shaded in grey are areas for which no data is available.


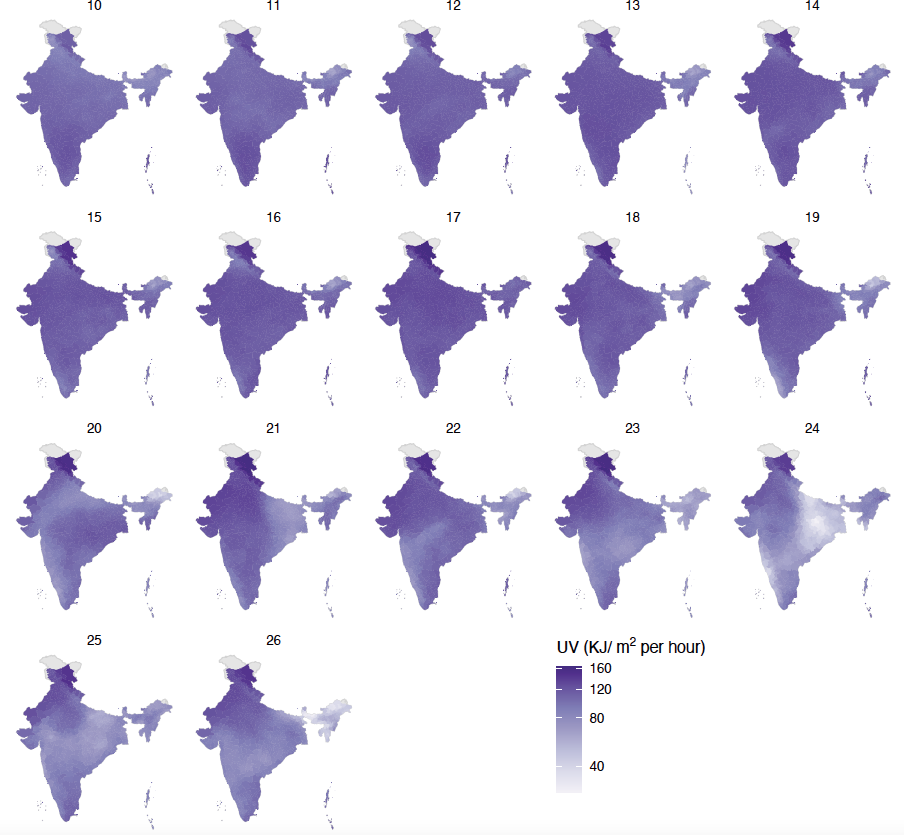


**S9 Fig**. Downward ultraviolet (UV) radiation (KJ/m2 per hour) during the Delta wave in India. The weeks in 2021 investigated are numbered in maps. Areas shaded in grey are areas for which no data is available.


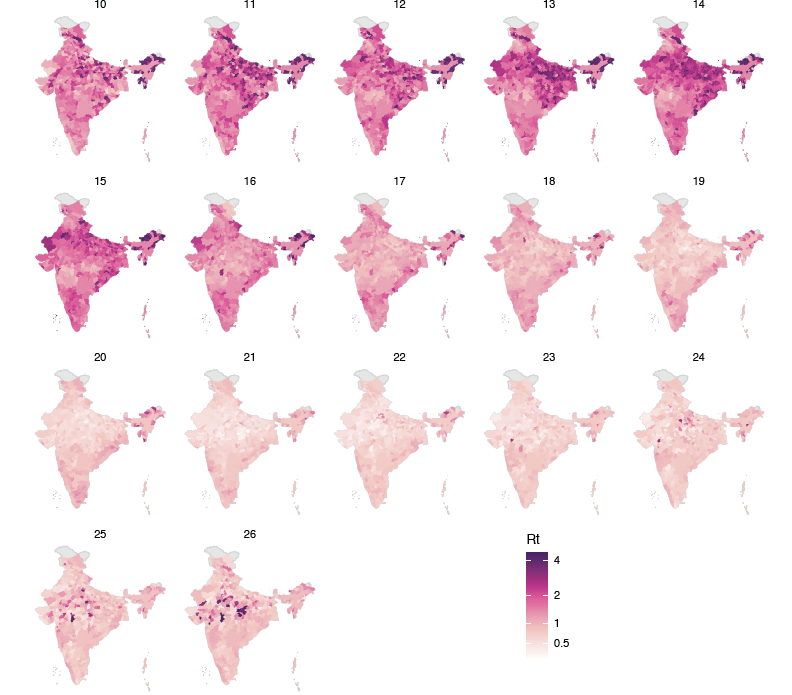


**S10 Fig**. Weekly Rt derived from COVID-19 cases reported during the Delta wave in India. The weeks in 2021 investigated are numbered in maps. Areas shaded in grey are areas for which no data is available.


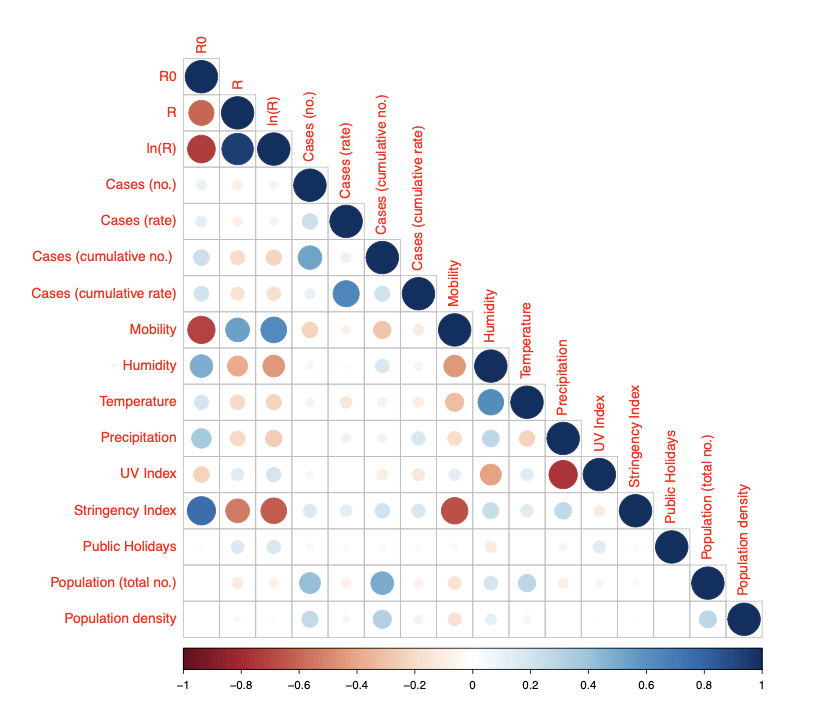


**S11 Fig**. Pairwise Pearson correlations between weekly means of variables at district level during the Delta wave in India, 2021. R0: basic reproduction number. Rt: instantaneous reproduction number. ln_R: log(Rt/R0). Cases_rate: new COVID-19 cases reported per 1000 people. Cases_accu_rate: cumulative cases per 1000 people reported since the first week of the wave. mean_intra: intra-district relative mobility. d2m: relative humidity. t2m: mean temperature of air (°C at 2m above the surface of land, sea or inland waters). tp: precipitation (metres). uv: downward ultraviolet radiation. Stringency: index of COVID-19 intervention stringency. Holiday: days of public holidays in a week. pop_sum: total population of each district. pop_density: population number per km2 of each district.


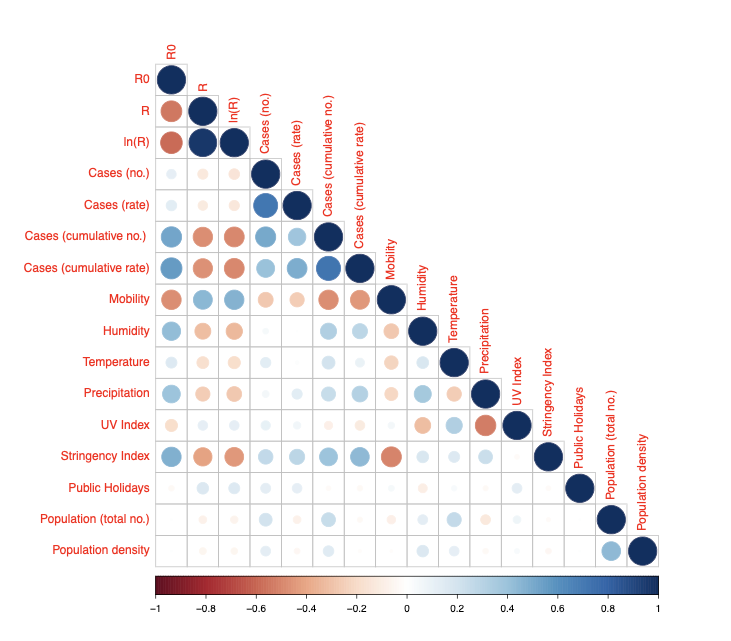


**S12 Fig**. Kendall rank correlations between weekly means of variables at district level during the Delta wave in India, 2021. R0: basic reproduction number. Rt: instantaneous reproduction number. ln_R: log(Rt/R0). Cases_rate: new COVID-19 cases reported per 1000 people. Cases_accu_rate: cumulative cases per 1000 people reported since the first week of the wave. mean_intra: intra-district relative mobility. d2m: relative humidity. t2m: mean temperature of air (°C at 2m above the surface of land, sea or inland waters). tp: precipitation (metres). uv: downward ultraviolet radiation. Stringency: index of COVID-19 intervention stringency. Holiday: days of public holidays in a week. pop_sum: total population of each district. pop_density: population number per km2 of each district.


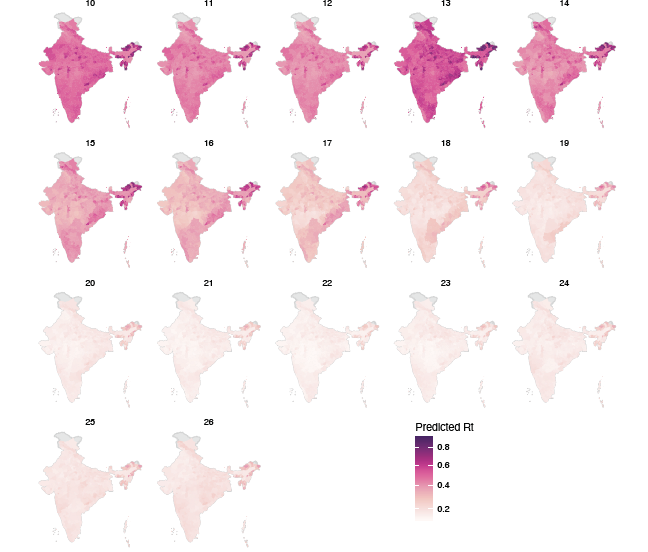


**S13 Fig**. Posterior predictive mean Rt during the Delta wave in India, 2021, derived from the best fitting model (model 4.1) at country level using leave-one-week-out cross-validation approach. The weeks in 2021 investigated are numbered in maps. Areas shaded in grey are areas for which no data is available.


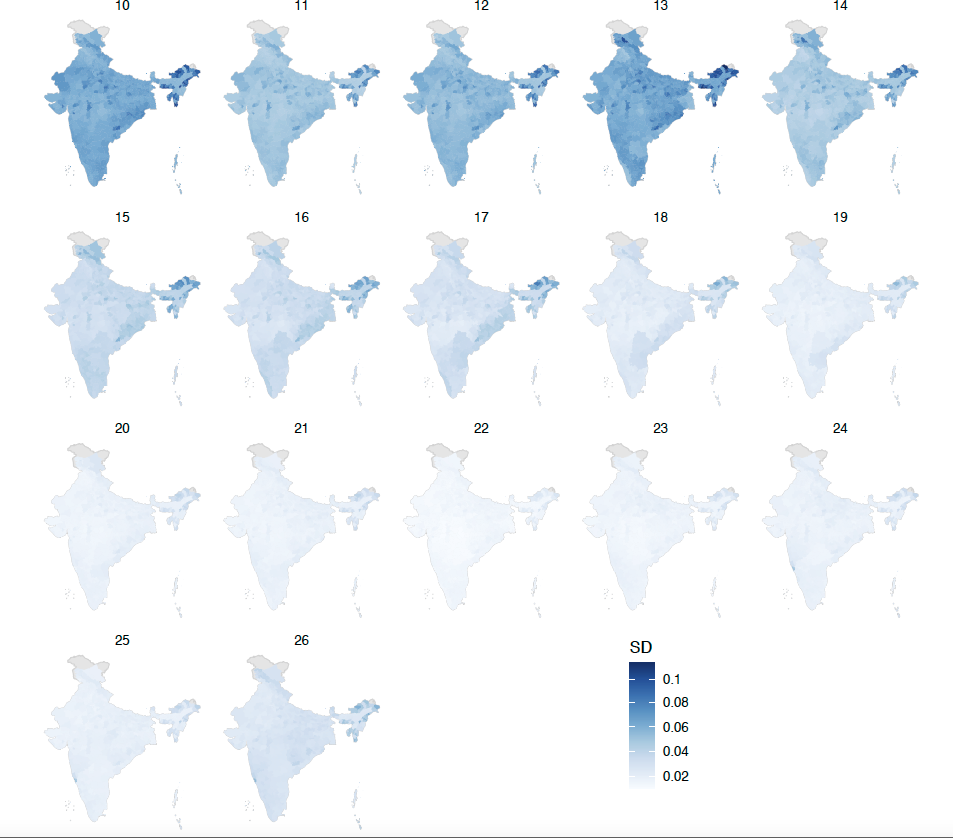


**S14 Fig**. Standard deviation (SD) of posterior predictive Rt during the Delta wave in India, 2021, derived from the best fitting model (model 4.1 without DLNMs) at country level using a leave-one-week-out cross-validation approach. Areas shaded in grey are areas for which no data is available.


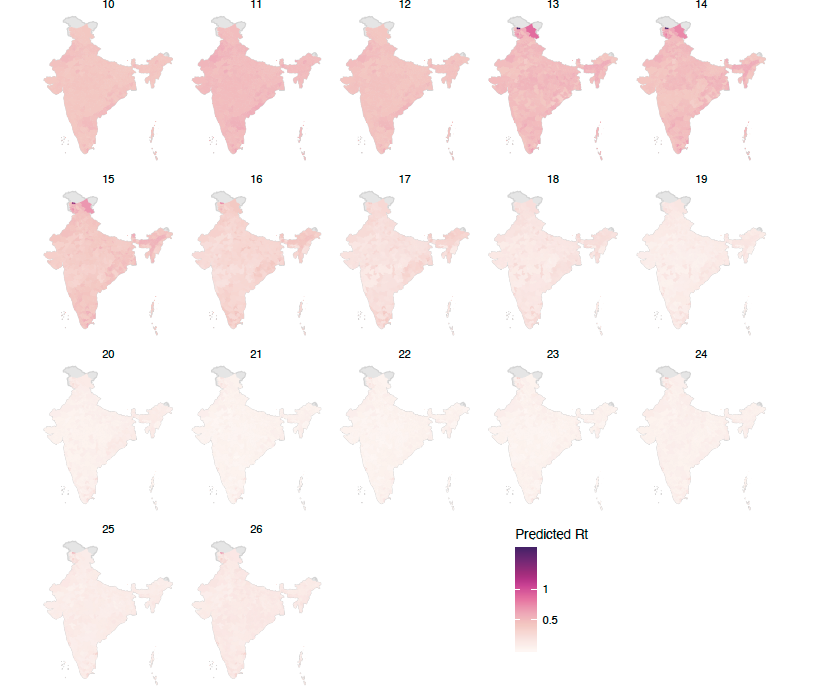


**S15 Fig**. Posterior predictive mean Rt during the Delta wave in India, 2021, derived from the best fitting model (model 4.1) at country level using leave-one-state-out cross-validation approach. The weeks in 2021 investigated are numbered in maps. Areas shaded in grey are areas for which no data is available.


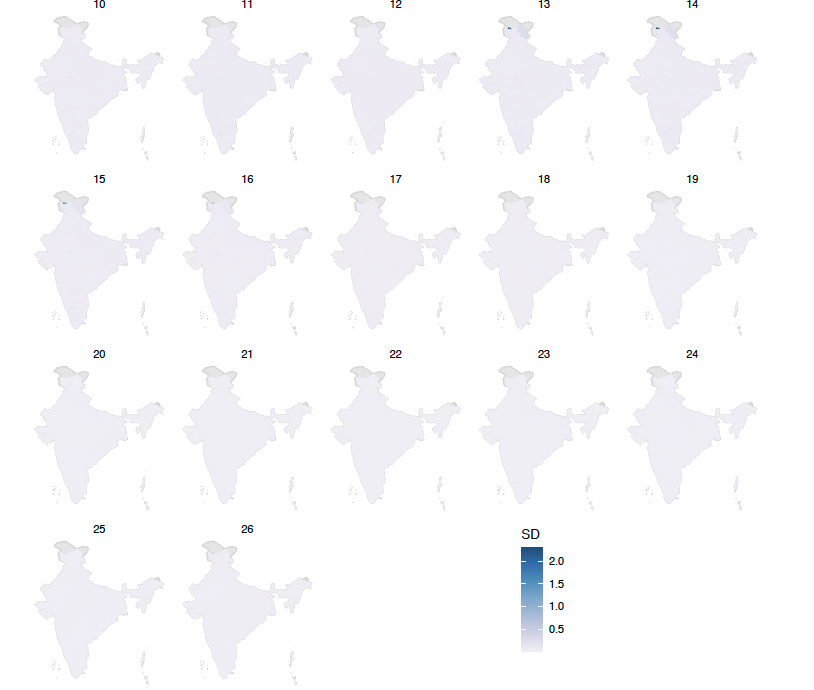


**S16 Fig**. Standard deviation (SD) of posterior predictive Rt during the Delta wave in India, 2021, derived from the best fitting model (model 4.1 without DLNMs) at country level using a leave-one-state-out cross-validation approach. Areas shaded in grey are areas for which no data is available.


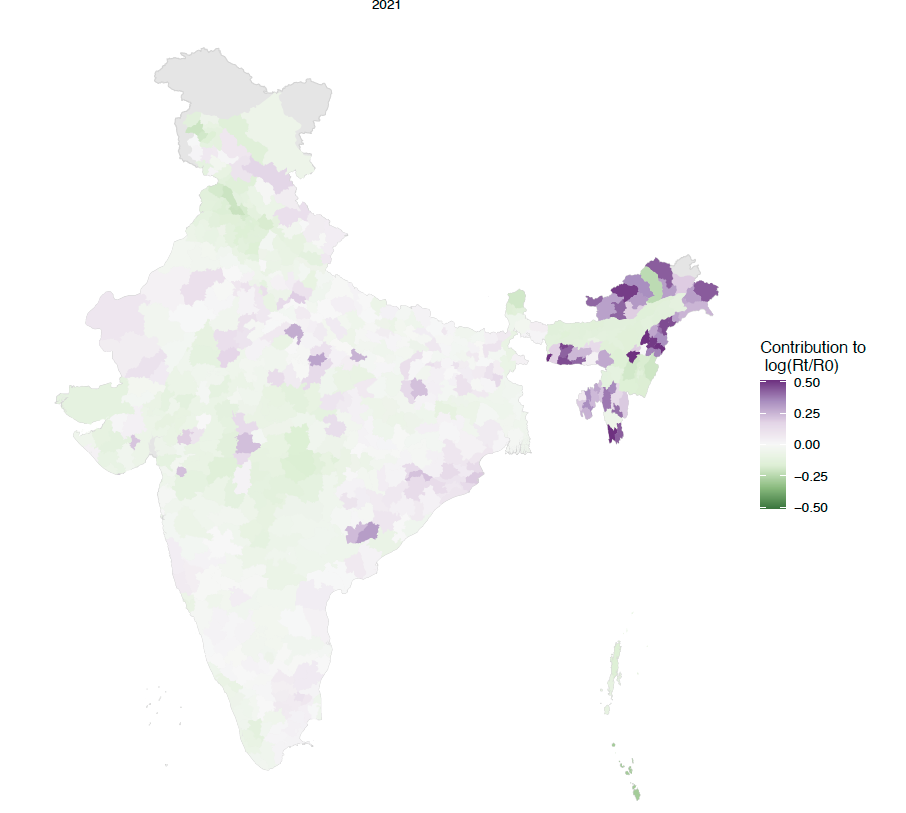


**S17 Fig**. Contribution of spatial random effects to estimates of Rt changes in the base model. Areas shaded in grey are areas for which no data is available.


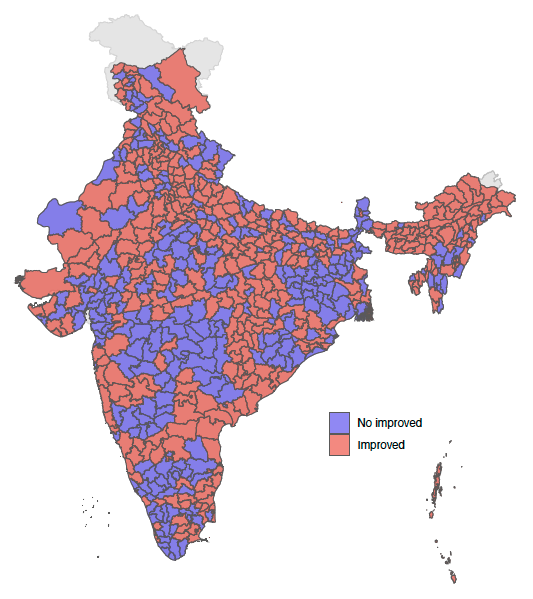

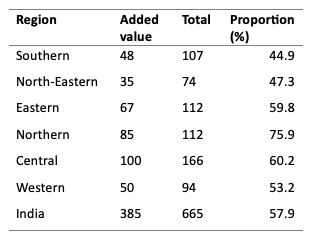


**S18 Fig**. Improvement by using the best fitting model across the country, compared to baseline model. Difference between mean absolute error (MAE) for the baseline model (weekly random effects, spatial random effects and population density) and MAE for the best fitting model (model 4.1 with DLNMs). Districts with positive values (pink) suggest that capturing the nonlinear and delayed impacts of mobility, climate information and intervention stringency, improves the model in these areas. Districts with negative values (blue) suggest that mobility, intervention and climate information did not improve the model fit and other unexplained factors might dominate space-time dynamics in these areas. The MAE of the selected model was smaller than the baseline model for 385 of the 665 (57.9%) districts in India, with the results of model performance provided by geo-political regions in the Table. Areas shaded in grey are areas for which no data is available.


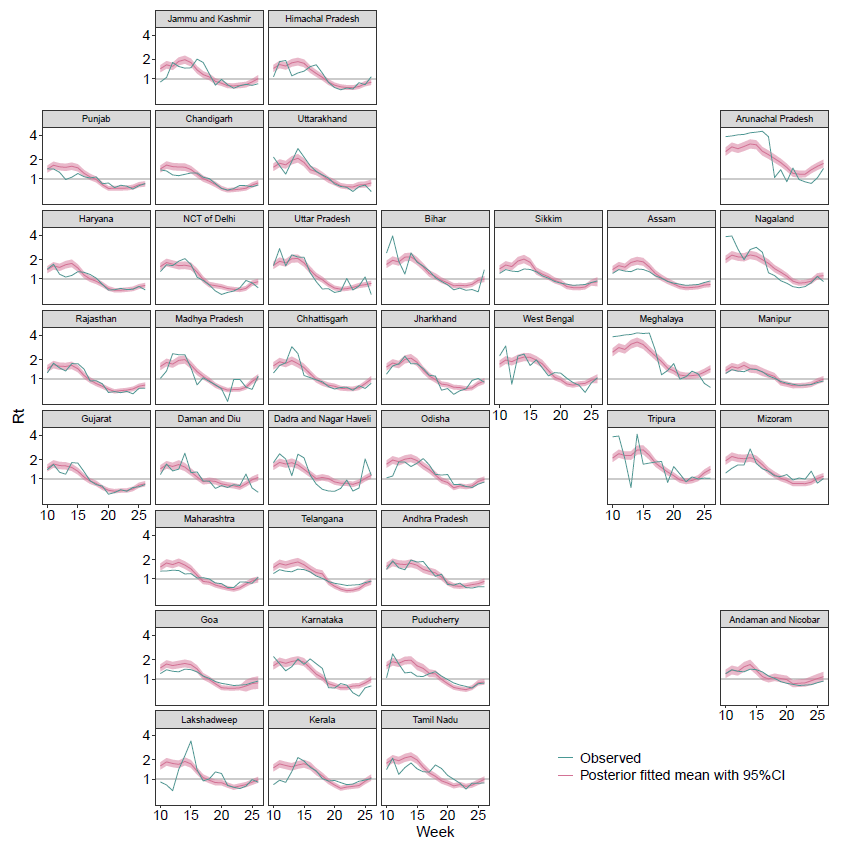


**S19 Fig.** Observed versus posterior fitted Rt in the capital district of each state using the best fitting model (model 4.1 with DLNMs) at country level. Graphs with a log scale at y-axis show the observed Rt derived from reported case data, and corresponding mean and 95% confidence interval (CI, shaded pink area) of fitted Rt, derived from the best fitting model (model 4.1 with DLNMs) at country level. States are ordered by their geographical location.


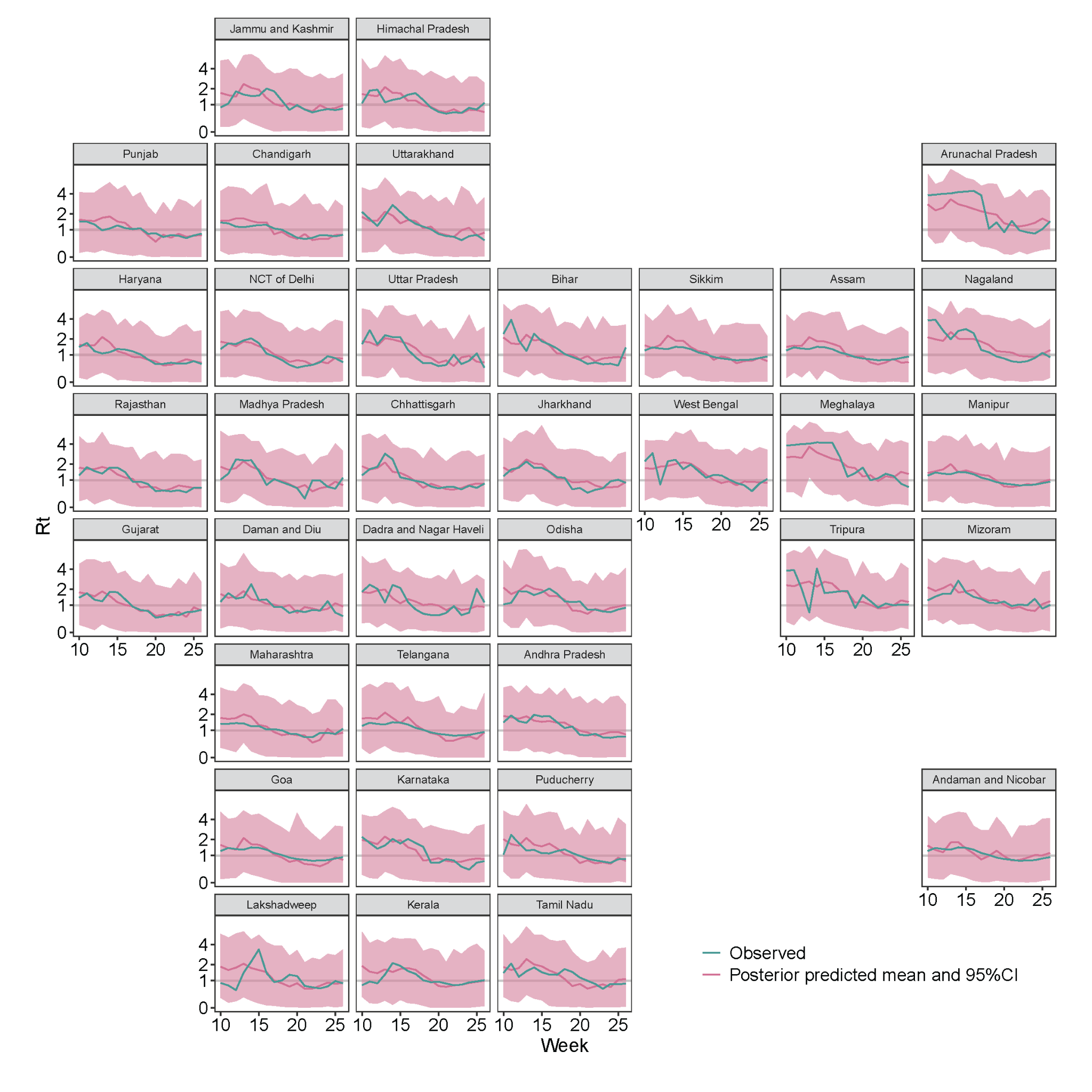


**S20 Fig.** Observed versus posterior predictive Rt in the capital district of each state, using leave-one-week-out cross-validation approach**.** Graphs with a log scale at y-axis show the observed Rt derived from reported case data, and corresponding posterior predictive mean and 95% prediction interval (CI, shaded pink area), derived from the best fitting model (model 4.1 with DLNMs) at country level. States are ordered by their geographical location.


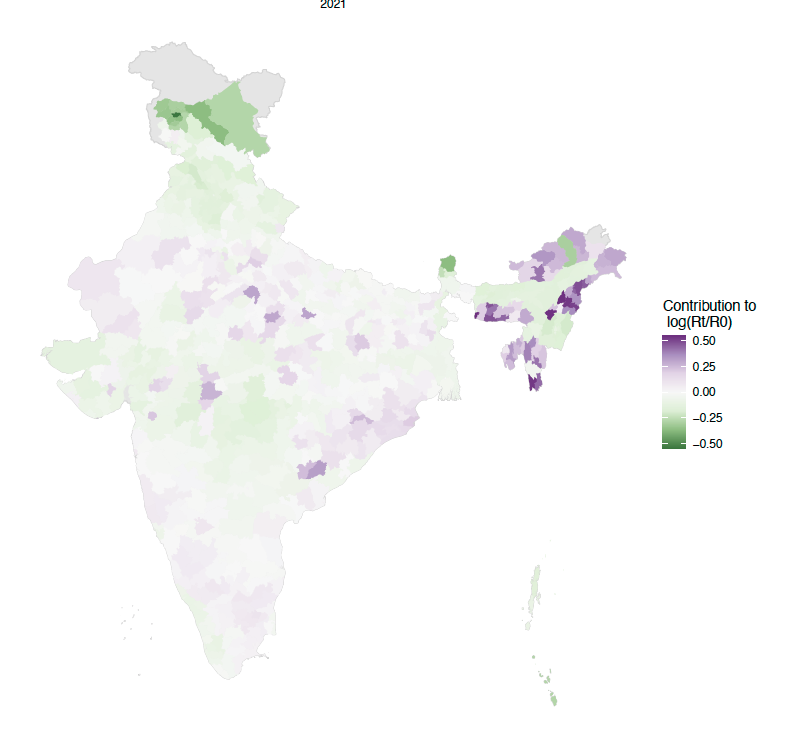


**S21 Fig**. Contribution of spatial random effects to estimates of Rt changes in the base model. Areas shaded in grey are areas for which no data is available.


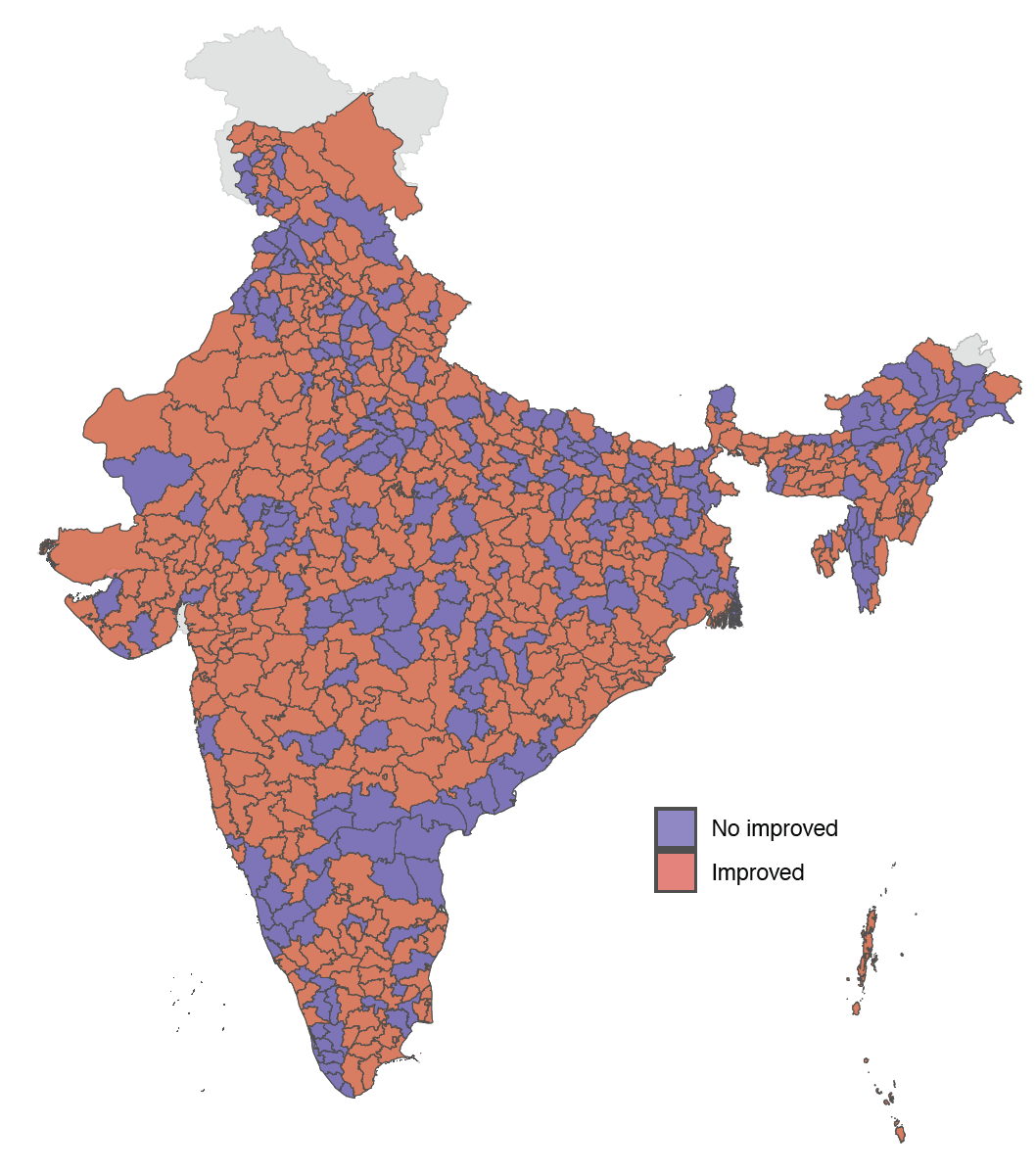


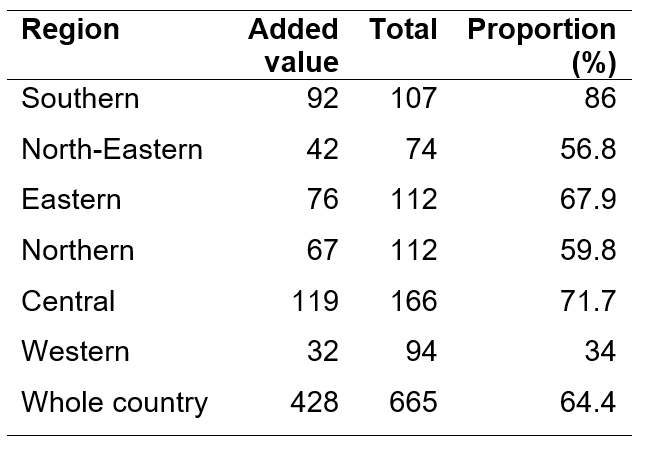


**S22 Fig**. Improvement of using the best fitting model with 2-week lag covariates (no DLNMs), compared to baseline model with the same lag. Difference between mean absolute error (MAE) for the baseline model and MAE for the best fitting model (Model 4.1). Districts with positive values (pink) suggest that capturing the 2-week lag impacts of mobility, temperature, UV and intervention stringency, improves the model in these areas. Districts with negative values (blue) suggest that mobility, intervention and climate information did not improve the model fit and other unexplained factors might dominate space-time dynamics in these areas. The MAE of the selected model was smaller than the baseline model for 428 of the 665 (64.4%) districts in India, and further improved the best fitting model with DLNMs (Fig S12). Results of model performance are provided by geo-political regions in the Table. Areas shaded in grey are areas for which no data is available.


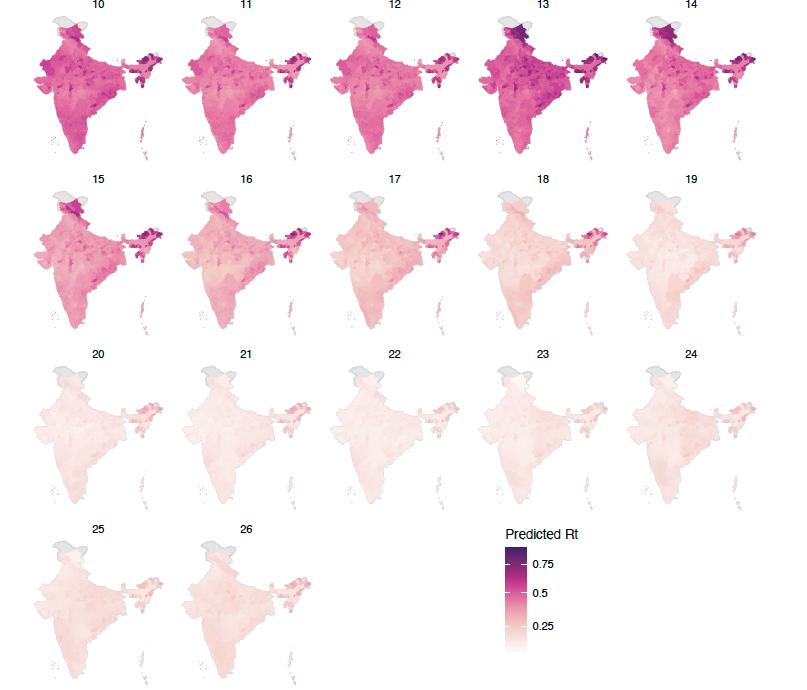


**S23 Fig**. Posterior predictive mean Rt during the Delta wave in India, 2021, derived from the best fitting model (model 4.1 without DLNMs) at country level using 2-week lag covariates and leave-one-week-out cross-validation approach. Areas shaded in grey are areas for which no data is available.


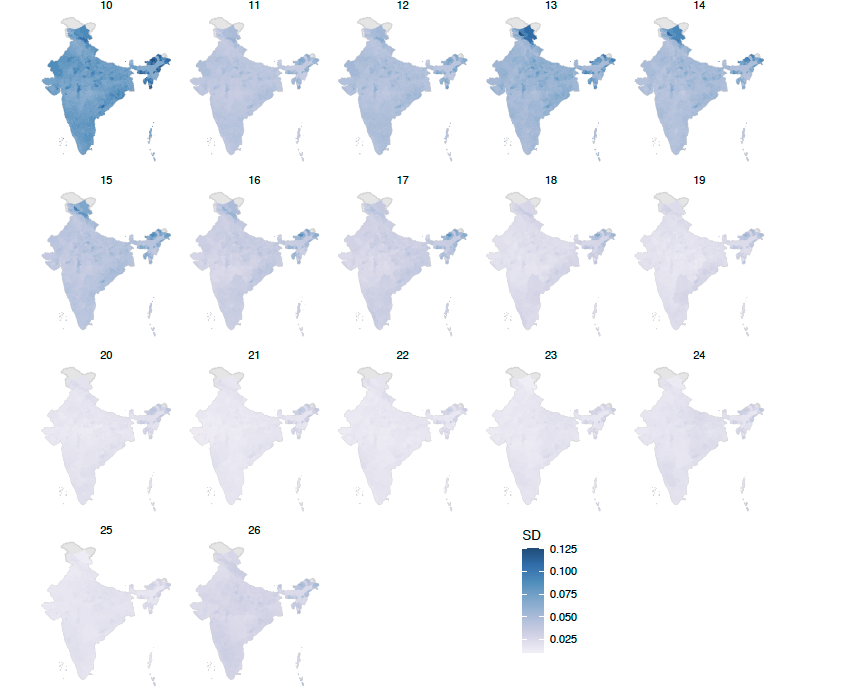


**S24 Fig**. Standard deviation (SD) of posterior predictive Rt during the Delta wave in India, 2021, derived from the best fitting model (model 4.1 without DLNMs) at country level using 2-week lag covariates and leave-one-week-out cross-validation approach. Areas shaded in grey are areas for which no data is available.


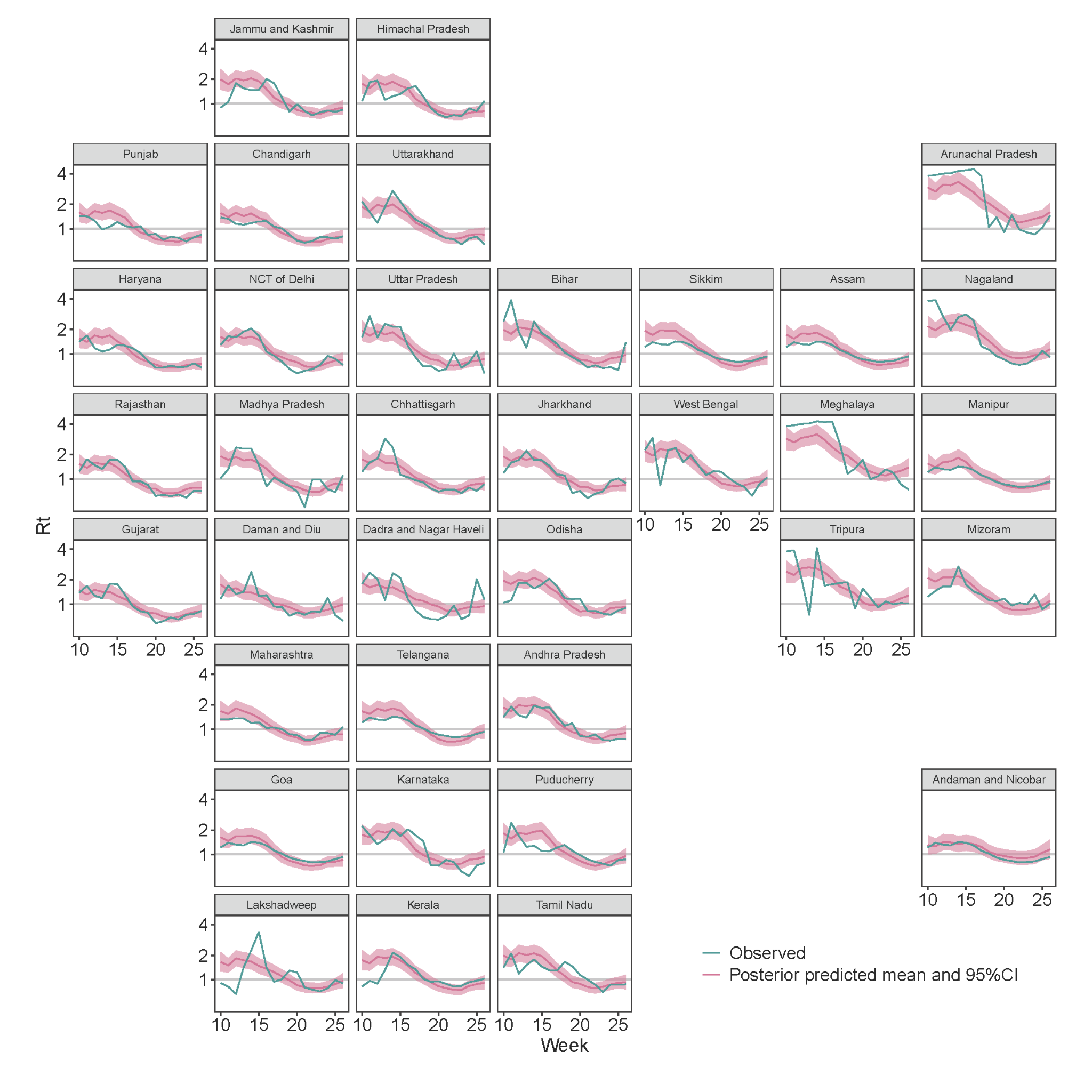


**S25 Fig. Observed versus posterior predictive Rt in the capital district of each state.** Graphs with a log scale at y-axis show the observed Rt derived from reported case data, and corresponding posterior predictive mean and 95% prediction interval (CI, shaded pink area), derived from the best fitting model without DLNMs at country level (model 4.1: base model + mobility + temperature + UV + intervention policy; see SI Table S2), using 2-week lag covariates and leave-one-week-out cross-validation approach. States are ordered by their geographical location.


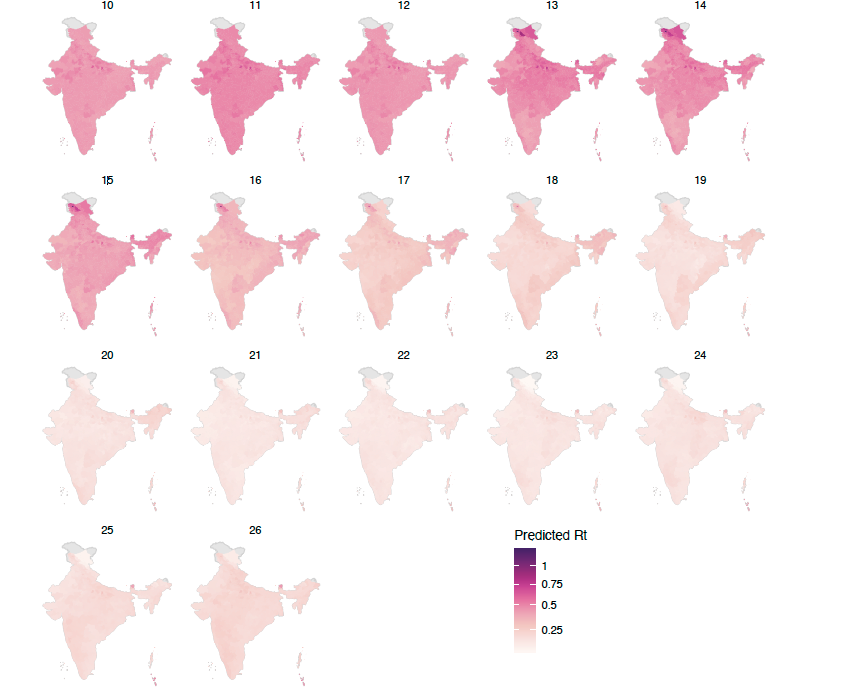


**S26 Fig**. Posterior predictive mean Rt during the Delta wave in India, 2021, derived from the best fitting model (model 4.1 without DLNMs) at country level using 2-week lag covariates and leave-one-state-out cross-validation approach. Areas shaded in grey are areas for which no data is available.


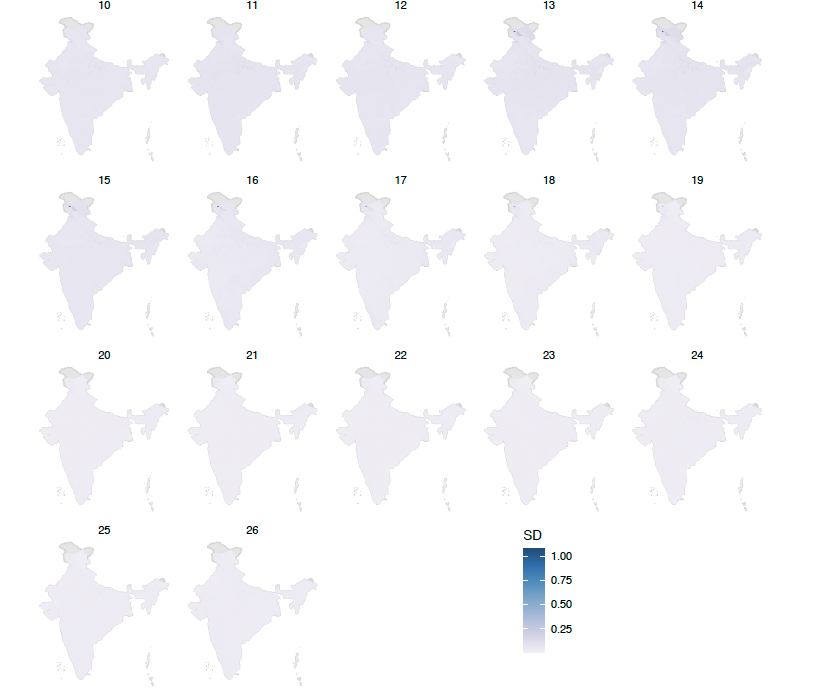


**S27 Fig**. Standard deviation (SD) of posterior predictive Rt during the Delta wave in India, 2021, derived from the best fitting model (model 4.1 without DLNMs) at country level using 2-week lag covariates and leave-one-state-out cross-validation approach. Areas shaded in grey are areas for which no data is available.

**Wave 1**

| **S5 Table. Wave 1: Adequacy results for models with DLNMs and increasing complexity.** | | | | | | | | | |
| --- | --- | --- | --- | --- | --- | --- | --- | --- | --- |
| Model | COVID-19 transmission risk Rt/R0 | Whole country | | Urban | | Suburban | | Rural | |
|  |  | DIC | LS | DIC | LS | DIC | LS | DIC | LS |
| Base model | Spatiotemporal random effects + D | 1512.50 | 0.058 | -803.51 | -0.070 | -6.524 | 0.001 | 1552.89 | 0.499 |
| 1.1 | Base model + M | 1483.01 | 0.059 | -854.45 | -0.069 | -16.33 | 0.000 | 1561.86 | 0.503 |
| 1.2 | Base model + T | 1422.88 | 0.056 | -852.07 | -0.075 | -38.35 | -0.002 | 1557.02 | 0.500 |
| 1.3 | Base model + P | 1461.12 | 0.057 | -843.46 | -0.074 | -37.34 | -0.002 | 1564.57 | 0.502 |
| 1.4 | Base model + S | 1514.22 | 0.059 | -803.25 | -0.070 | -4.14 | 0.001 | 1559.95 | 0.501 |
| 2.1 | Base model + M + T | 1394.91 | 0.056 | -905.17 | -0.074 | -57.71 | -0.003 | 1561.74 | 0.503 |
| 2.2 | Base model + M + P | 1445.66 | 0.057 | -889.86 | -0.073 | -52.02 | -0.003 | 1571.19 | 0.505 |
| 2.3 | Base model + M + S | 1484.39 | 0.059 | -852.99 | -0.069 | -18.89 | 0.000 | 1567.35 | 0.505 |
| 2.4 | Base model + M + H | 1476.51 | 0.059 | -858.00 | -0.070 | -17.09 | 0.000 | 1563.32 | 0.503 |
| 3.1 | Base model + M + T + P | 1367.46 | 0.055 | -939.30 | -0.077 | -74.29 | -0.004 | 1574.42 | 0.507 |
| 3.2 | Base model + M + T + S | 1388.82 | 0.056 | -906.18 | -0.074 | -52.14 | -0.003 | 1569.75 | 0.506 |
| 3.3 | Base model + M + T + H | 1391.73 | 0.056 | -905.49 | -0.074 | -57.39 | -0.003 | 1563.06 | 0.503 |
| 3.4 | Base model + M + P + S | 1425.17 | 0.057 | -892.36 | -0.073 | -55.88 | -0.003 | 1576.71 | 0.507 |
| 3.5 | Base model + M + P + H | 1419.90 | 0.057 | -890.78 | -0.073 | -53.54 | -0.003 | 1572.21 | 0.506 |
| 3.6 | Base model + M + S + H | 1480.03 | 0.059 | -854.83 | -0.070 | -13.40 | 0.001 | 1567.68 | 0.505 |
| 4.1 | Base model + M + T + P + S | 1363.04 | 0.055 | -939.04 | -0.077 | -68.30 | -0.004 | 1580.66 | 0.509 |
| 4.2 | Base model + M + T + P + H | 1366.68 | 0.055 | -940.27 | -0.077 | -75.09 | -0.005 | 1575.53 | 0.507 |
| 4.3 | Base model + M + T + S + H | 1390.20 | 0.056 | -904.08 | -0.074 | -51.12 | -0.002 | 1570.53 | 0.506 |
| 4.4 | Base model + M + P + S + H | 1423.03 | 0.057 | -890.50 | -0.073 | -53.81 | -0.003 | 1577.33 | 0.507 |

DIC: deviance information criterion; LS: logarithmic score; D: cumulative cases per 1000 people since the first week in the modelling; M: mobility; D: humidity; T: temperature; U: ultraviolet radiation; S: stringency index of intervention policy; H: public holidays. *DIC of the best fitting model for each region.

**S6 Table. Wave 1: Adequacy results for models (without DLNMs) using 2-week lag covariates with increasing complexity.**

| Model | COVID-19 transmission risk Rt/R0 | Whole country | | Urban | | Suburban | | Rural | |
| --- | --- | --- | --- | --- | --- | --- | --- | --- | --- |
|  |  | DIC | LS | DIC | LS | DIC | LS | DIC | LS |
| Base model | Spatiotemporal random effects + D | 1519.54 | 0.059 | -800.60 | -0.070 | 3.114 | 0.002 | 1552.15 | 0.498 |
| 1.1 | Base model + M | 1492.67 | 0.058 | -808.29 | -0.071 | -1.43 | 0.001 | 1553.74 | 0.499 |
| 1.2 | Base model + T | 1478.85 | 0.058 | -808.75 | -0.071 | -23.84 | -0.001 | 1551.78 | 0.498 |
| 1.3 | Base model + P | 1459.63 | 0.057 | -834.85 | -0.073 | -28.05 | -0.001 | 1546.67 | 0.497 |
| 1.4 | Base model + S | 1524.38 | 0.059 | -802.47 | -0.071 | 0.80 | 0.002 | 1551.98 | 0.499 |
| 2.1 | Base model + M + T | 1454.84 | 0.057 | -819.55 | -0.072 | -26.17 | -0.001 | 1552.93 | 0.499 |
| 2.2 | Base model + M + P | 1428.88 | 0.056 | -846.71 | -0.074 | -32.13 | -0.001 | 1549.03 | 0.498 |
| 2.3 | Base model + M + S | 1493.18 | 0.058 | -812.00 | -0.071 | 0.42 | 0.001 | 1553.68 | 0.499 |
| 2.4 | Base model + M + H | 1493.71 | 0.058 | -819.61 | -0.072 | -1.65 | 0.001 | 1552.89 | 0.499 |
| 3.1 | Base model + M + T + P | 1426.92 | 0.055 | -844.46 | -0.074 | -39.02 | -0.002 | 1550.99 | 0.498 |
| 3.2 | Base model + M + T + S | 1448.50 | 0.056 | -818.86 | -0.072 | -24.79 | -0.001 | 1553.27 | 0.499 |
| 3.3 | Base model + M + T + H | 1457.08 | 0.057 | -820.15 | -0.072 | -26.87 | -0.001 | 1553.11 | 0.499 |
| 3.4 | Base model + M + P + S | 1430.20 | 0.056 | -845.50 | -0.074 | -32.16 | -0.001 | 1549.50 | 0.498 |
| 3.5 | Base model + M + P + H | 1427.84 | 0.056 | -846.64 | -0.074 | -32.87 | -0.001 | 1549.73 | 0.498 |
| 3.6 | Base model + M + S + H | 1493.68 | 0.058 | -811.29 | -0.071 | 0.26 | 0.001 | 1553.97 | 0.499 |
| 4.1 | Base model + M + T + P + S | 1420.55 | 0.055 | -844.35 | -0.074 | -39.05 | -0.002 | 1551.69 | 0.499 |
| 4.2 | Base model + M + T + P + H | 1427.78 | 0.055 | -845.57 | -0.074 | -40.35 | -0.002 | 1551.66 | 0.499 |
| 4.3 | Base model + M + T + S + H | 1454.32 | 0.057 | -819.20 | -0.072 | -26.43 | -0.001 | 1553.65 | 0.499 |
| 4.4 | Base model + M + P + S + H | 1429.82 | 0.056 | -848.73 | -0.074 | -33.81 | -0.001 | 1549.89 | 0.498 |

DIC: deviance information criterion; LS: logarithmic score; D: cumulative cases per 1000 people since the first week in the modelling; M: mobility; D: humidity; T: temperature; U: ultraviolet radiation; S: stringency index of intervention policy; H: public holidays. *DIC of the best fitting model for each region.

**Hyperparameters**

| **S7 Table. Model hyperparameters using a range of prior distributions in best fit model 4.1 for Wave 1** | | | | | | | |
| --- | --- | --- | --- | --- | --- | --- | --- |
| **Prior distributions** | **Model evaluation** | **Mean** | **SD** | **0.025 quantile** | **0.5 quantile** | **0.975 quantile** | **DIC** |
| $u=3\cdot SD$  $\alpha=0.01$ | Precision for base model | 17.309 | 0.210 | 1.863 | 17.321 | 17.686 | 1378.23 |
|  | Precision for rw1 | 83.388 | 62.752 | 21.715 | 66.041 | 251.721 |  |
|  | Precision for BYM2 | 111.929 | 9.220 | 97.319 | 110.797 | 133.265 |  |
|  |  |  |  |  |  |  |  |
| $u=2\cdot SD$  $\alpha=0.01$ | Precision for base model | 17.307 | 0.209 | 16.862 | 17.320 | 17.683 | 1378.11 |
|  | Precision for rw1 | 84.687 | 63.651 | 22.502 | 67.050 | 255.509 |  |
|  | Precision for BYM2 | 112.027 | 0.054 | 0.028 | 0.069 | 0.234 |  |
|  |  |  |  |  |  |  |  |
| $u=SD$  $\alpha=0.01$ | Precision for base model | 17.33 | 0.215 | 16.857 | 17.344 | 17.693 | 1380.60 |
|  | Precision for rw1 | 85.81 | 65.063 | 24.672 | 67.572 | 261.141 |  |
|  | Precision for BYM2 | 104.21 | 8.784 | 85.508 | 104.695 | 119.632 |  |
|  |  |  |  |  |  |  |  |
| $u=0.5$  $\alpha=0.001$ | Precision for base model | 17.33 | 0.216 | 16.857 | 17.346 | 17.696 | 1380.94 |
|  | Precision for rw1 | 86.19 | 65.354 | 24.832 | 67.846 | 262.229 |  |
|  | Precision for BYM2 | 104.51 | 8.568 | 86.298 | 104.971 | 119.615 |  |
|  |  |  |  |  |  |  |  |
| $u=0.5$  $\alpha=0.01$ | Precision for base model | 17.325 | 0.212 | 16.860 | 17.343 | 17.686 | 1377.56 |
|  | Precision for rw1 | 84.242 | 63.439 | 24.197 | 66.526 | 255.319 |  |
|  | Precision for BYM2 | 103.318 | 9.511 | 83.006 | 103.841 | 119.816 |  |
|  |  |  |  |  |  |  |  |
| $u=0.5$  $\alpha=0.1$ | Precision for base model | 17.309 | 0.210 | 16.863 | 17.321 | 17.686 | 1378.18 |
|  | Precision for rw1 | 83.579 | 62.855 | 21.848 | 66.199 | 252.204 |  |
|  | Precision for BYM2 | 111.949 | 9.229 | 97.333 | 110.814 | 133.314 |  |
|  |  |  |  |  |  |  |  |
| $u=1$  $\alpha=0.001$ | Precision for base model | 17.306 | 0.209 | 16.860 | 17.319 | 17.681 | 1378.00 |
|  | Precision for rw1 | 85.008 | 63.980 | 22.629 | 67.269 | 256.782 |  |
|  | Precision for BYM2 | 112.075 | 9.273 | 97.447 | 110.920 | 113.587 |  |
|  |  |  |  |  |  |  |  |
| $u=0.1$  $\alpha=0.01$ | Precision for base model | 17.267 | 0.198 | 16.828 | 17.284 | 17.601 | 1364.86 |
|  | Precision for rw1 | 110.706 | 88.347 | 34.930 | 85.528 | 349.537 |  |
|  | Precision for BYM2 | 116.200 | 12.907 | 98.191 | 114.169 | 147.760 |  |
|  |  |  |  |  |  |  |  |
| $u=0.1$  $\alpha=0.001$ | Precision for base model | 17.385 | 0.219 | 16.946 | 17.388 | 17.810 | 1362.94 |
|  | Precision for rw1 | 182.601 | 117.178 | 22.759 | 161.245 | 440.005 |  |
|  | Precision for BYM2 | 107.229 | 8.662 | 90.247 | 107.260 | 124.254 |  |
| DIC: deviance information criterion. *DIC of the best fitting model (model 4.1) for each region. rw1 is the temporal random effect model, $r_{t}$; BYM2 is the spatial random effect model, $b_{i}$; SD=0.36 standard deviation of $\Delta R_{t}$ during the Wave 1. | | | | | | | |


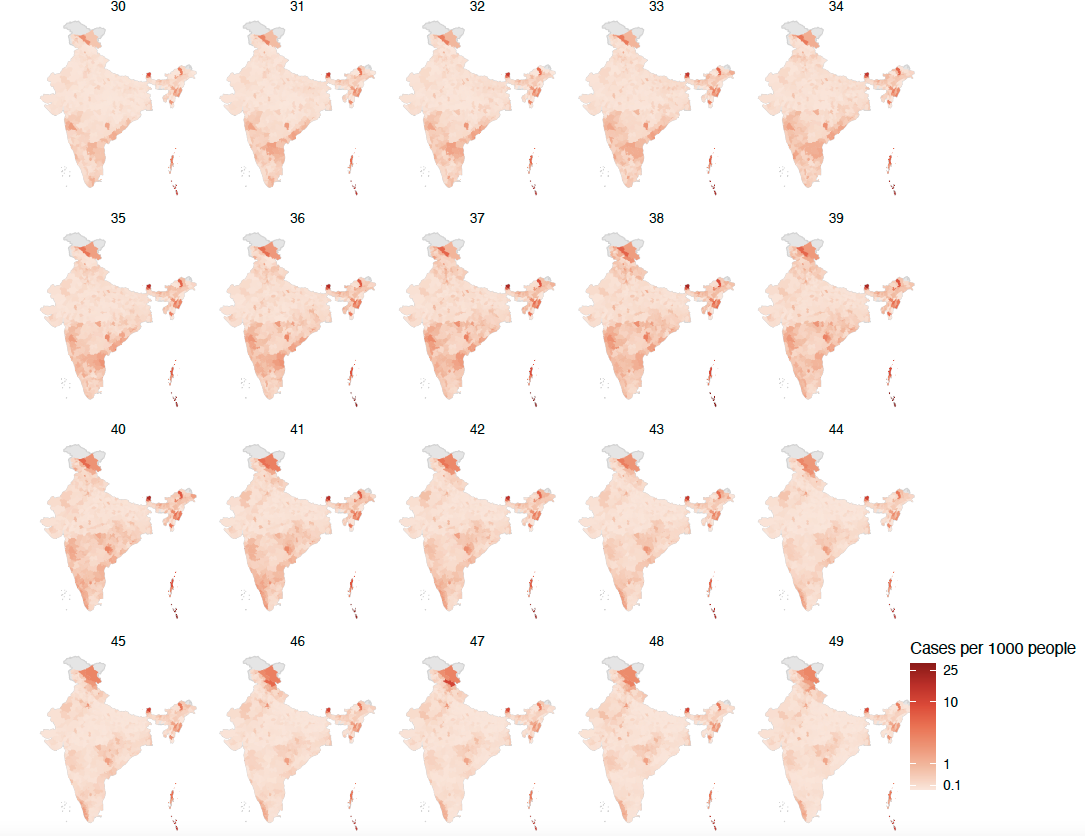


**S28 Fig**. COVID-19 cases reported by district each week during wave 1 in India. The weeks in 2020 investigated are numbered in maps. Areas shaded in grey are areas for which no data is available.


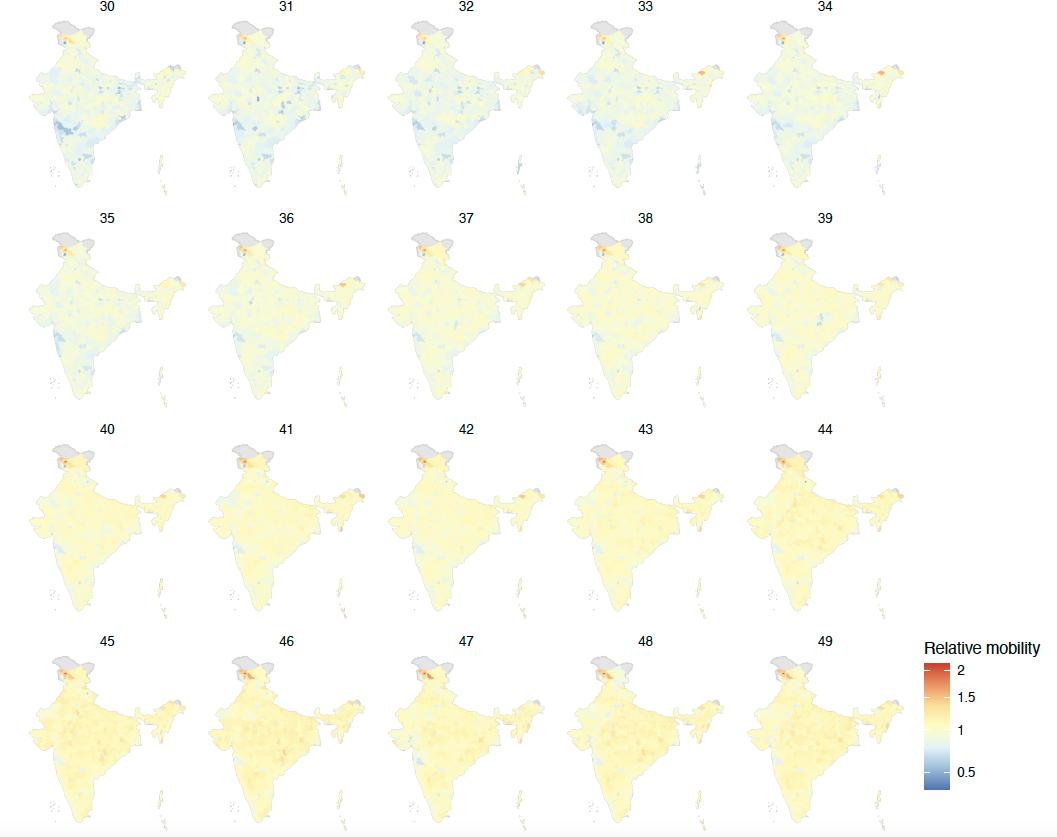


**S29 Fig**. Relative intra-district mobility during wave 1 in India, standardised by pre-pandemic mean baseline levels of mobility for the first eight weeks of 2020 (December 29, 2019 – February 22, 2020) for each district. The weeks in 2020 investigated are numbered in maps. Areas shaded in grey are areas for which no data is available.


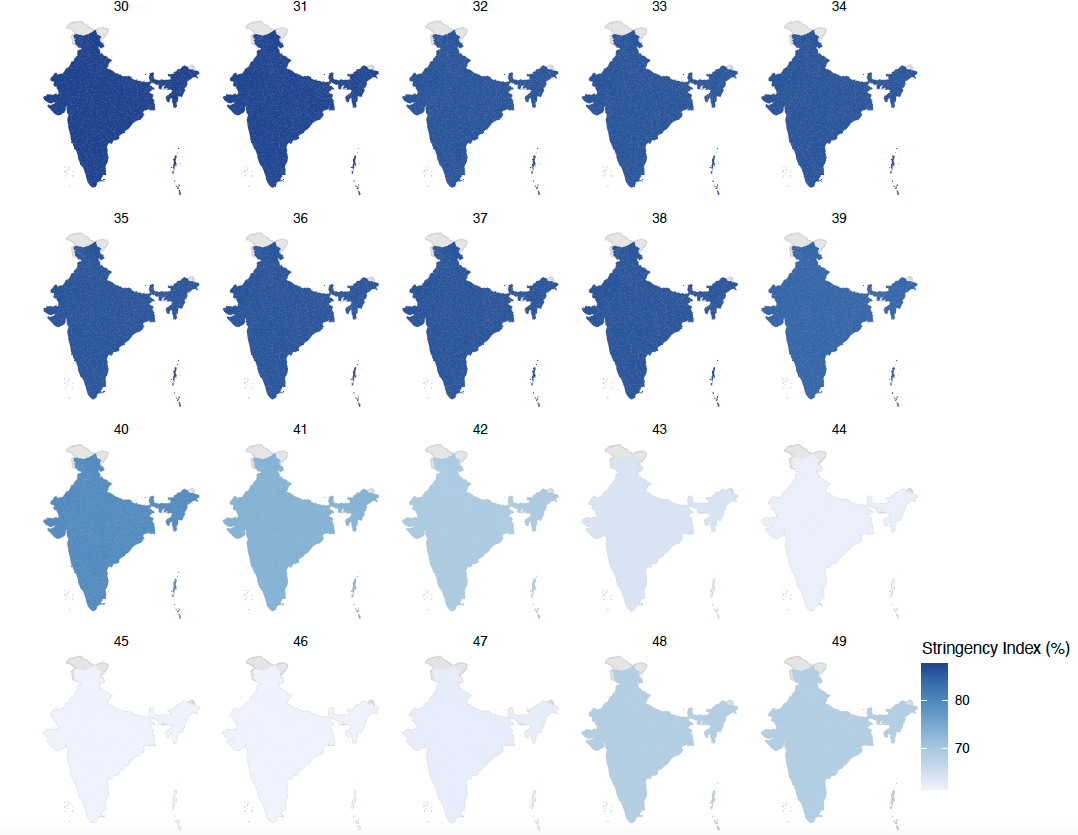


**S30 Fig**. Stringency Index of COVID-19 intervention policy implemented during wave 1 in India. The weeks in 2020 investigated are numbered in maps. Areas shaded in grey are areas for which no data is available.


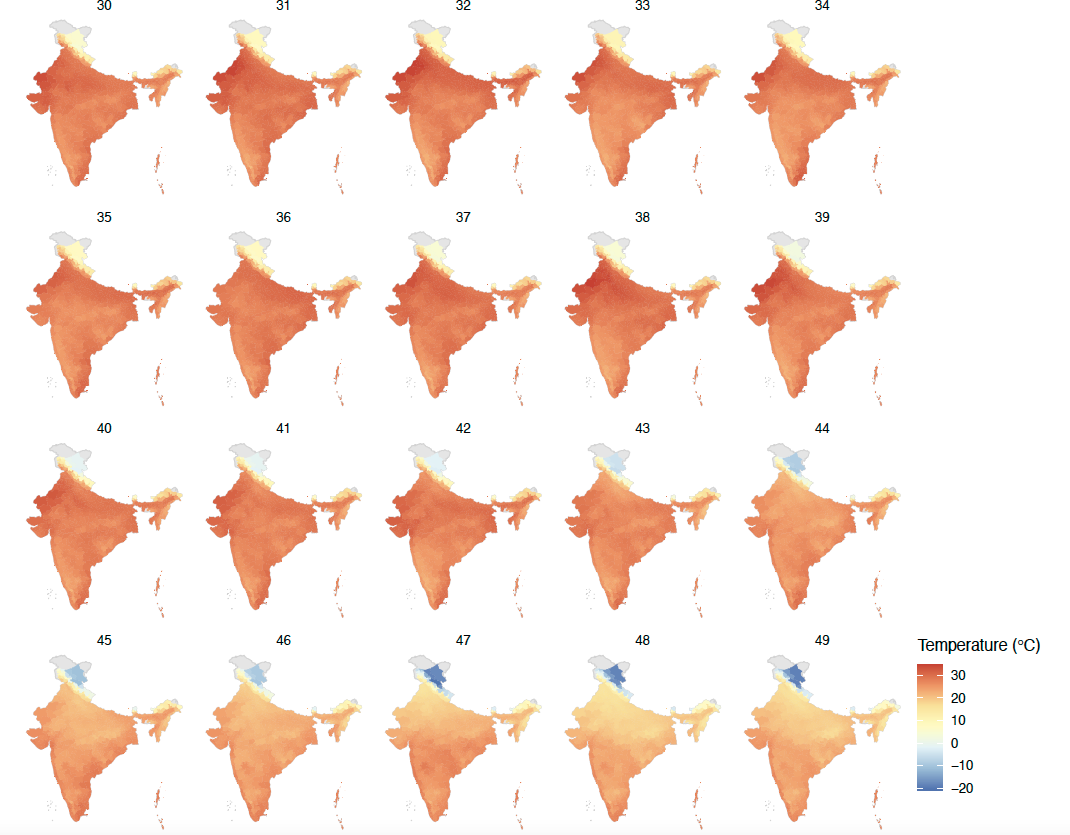


**S31 Fig**. Mean temperature at 2m above the surface during wave 1 in India. The weeks in 2020 investigated are numbered in maps. Areas shaded in grey are areas for which no data is available.


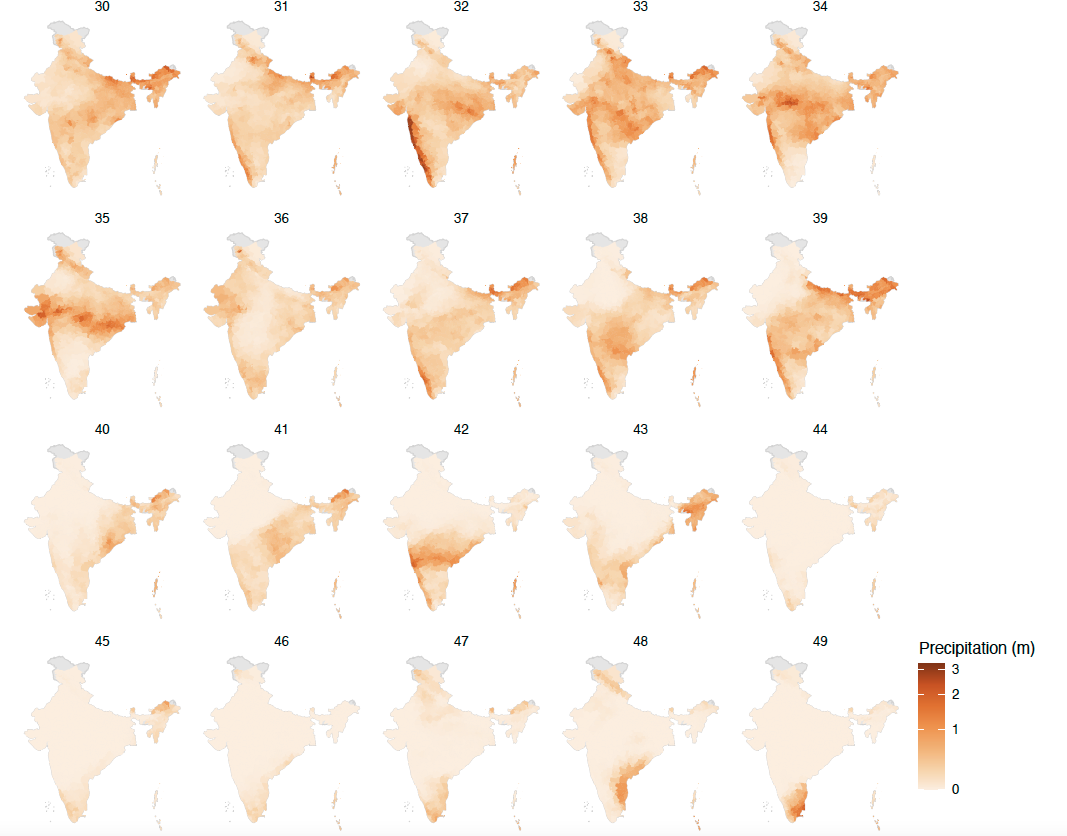


**S32 Fig**. Accumulated weekly precipitation (metres) during wave 1 in India. The weeks in 2020 investigated are numbered in maps. Areas shaded in grey are areas for which no data is available.


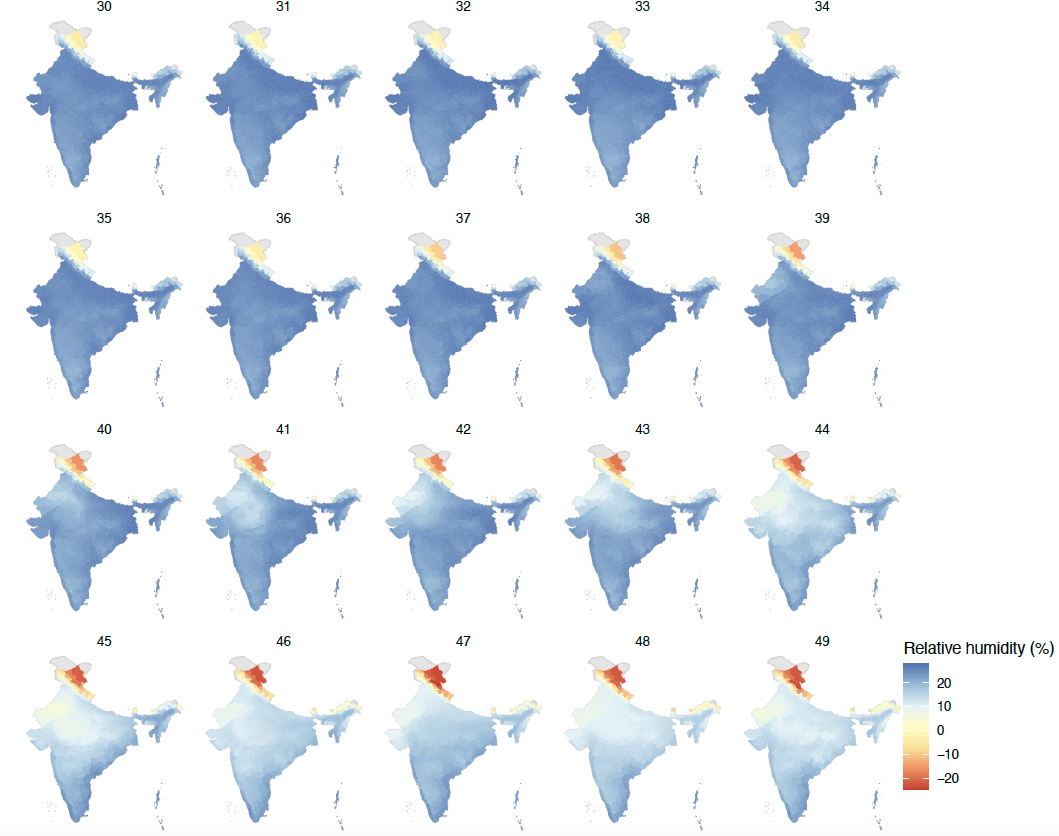


**S33 Fig**. Relative humidity during wave 1 in India. The weeks in 2020 investigated are numbered in maps. Areas shaded in grey are areas for which no data is available.


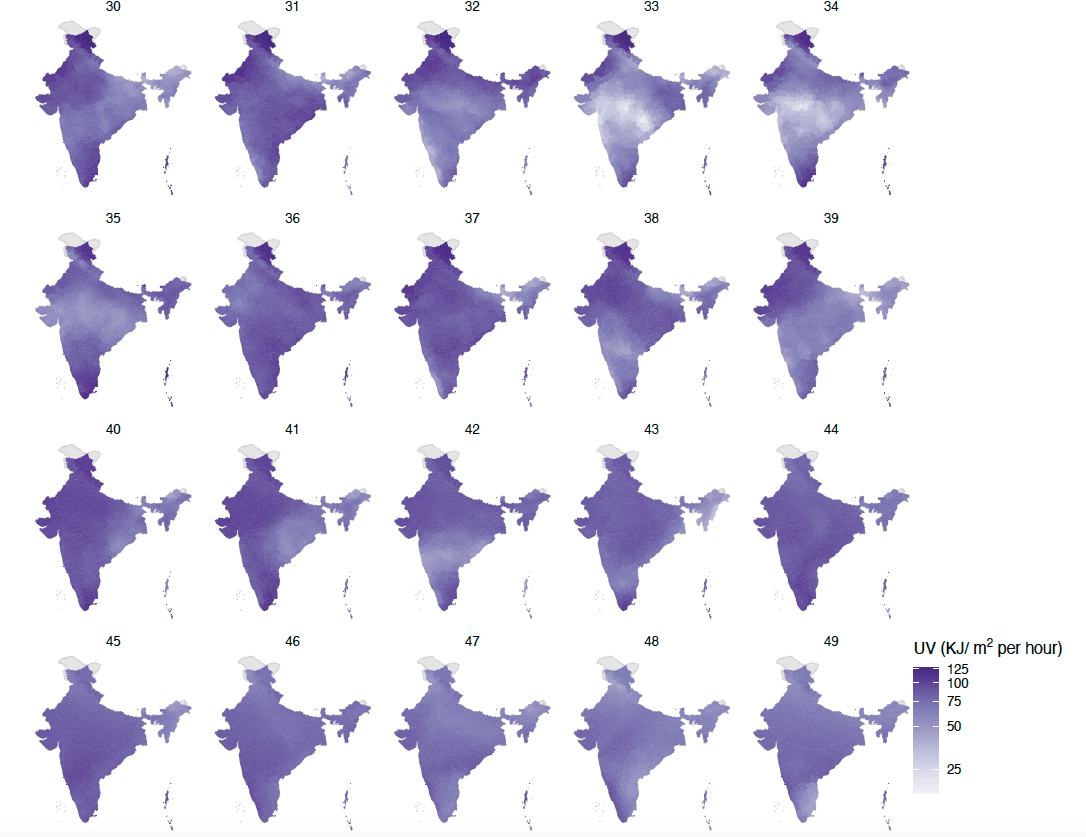


**S34 Fig**. Downward ultraviolet (UV) radiation (KJ/m2 per hour) during wave 1 in India. The weeks in 2020 investigated are numbered in maps. Areas shaded in grey are areas for which no data is available.


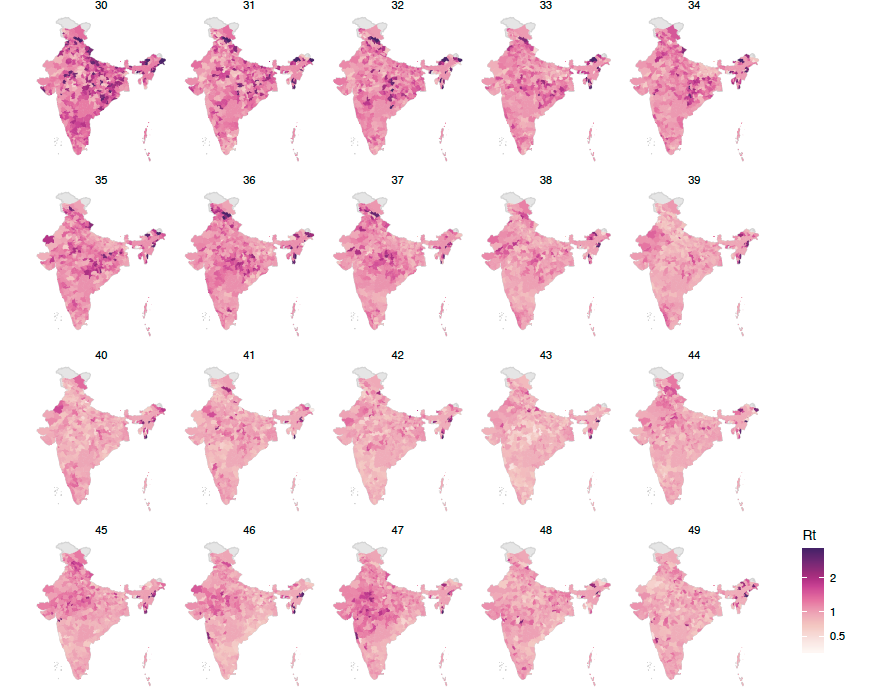


**S35 Fig**. Weekly Rt derived from COVID-19 cases reported during the wave 1 in India. The weeks in 2020 investigated are numbered in maps. Areas shaded in grey are areas for which no data is available.


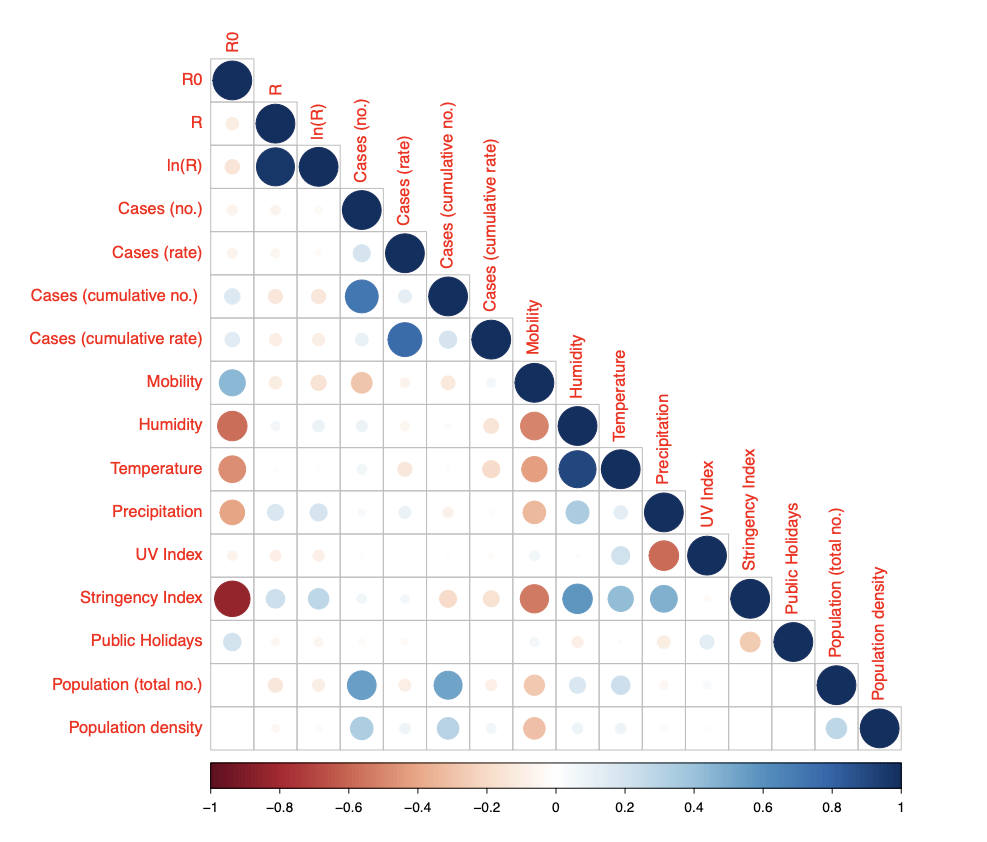


**S36 Fig.** Pairwise Pearson correlations between weekly means of variables at district level during the wave 1 in India, 2020. R0: basic reproduction number. Rt: instantaneous reproduction number. ln_R: log(Rt/R0). Cases_rate: new COVID-19 cases reported per 1000 people. Cases_accu_rate: cumulative cases per 1000 people reported since the first week of the wave. mean_intra: intra-district relative mobility. d2m: relative humidity. t2m: mean temperature of air (°C at 2m above the surface of land, sea or inland waters). tp: precipitation (metres). uv: downward ultraviolet radiation. Stringency: index of COVID-19 intervention stringency. Holiday: days of public holidays in a week. pop_sum: total population of each district. pop_density: population number per km2 of each district.


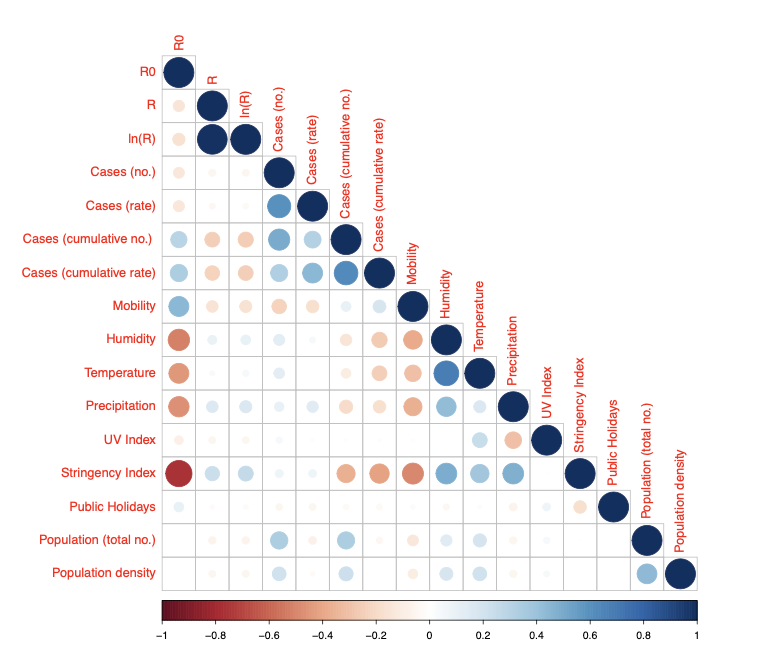


**S37 Fig.** Kendall rank correlations between weekly means of variables at district level during the wave 1 in India, 2020. R0: basic reproduction number. Rt: instantaneous reproduction number. ln_R: log(Rt/R0). Cases_rate: new COVID-19 cases reported per 1000 people. Cases_accu_rate: cumulative cases per 1000 people reported since the first week of the wave. mean_intra: intra-district relative mobility. d2m: relative humidity. t2m: mean temperature of air (°C at 2m above the surface of land, sea or inland waters). tp: precipitation (metres). uv: downward ultraviolet radiation. Stringency: index of COVID-19 intervention stringency. Holiday: days of public holidays in a week. pop_sum: total population of each district. pop_density: population number per km2 of each district.


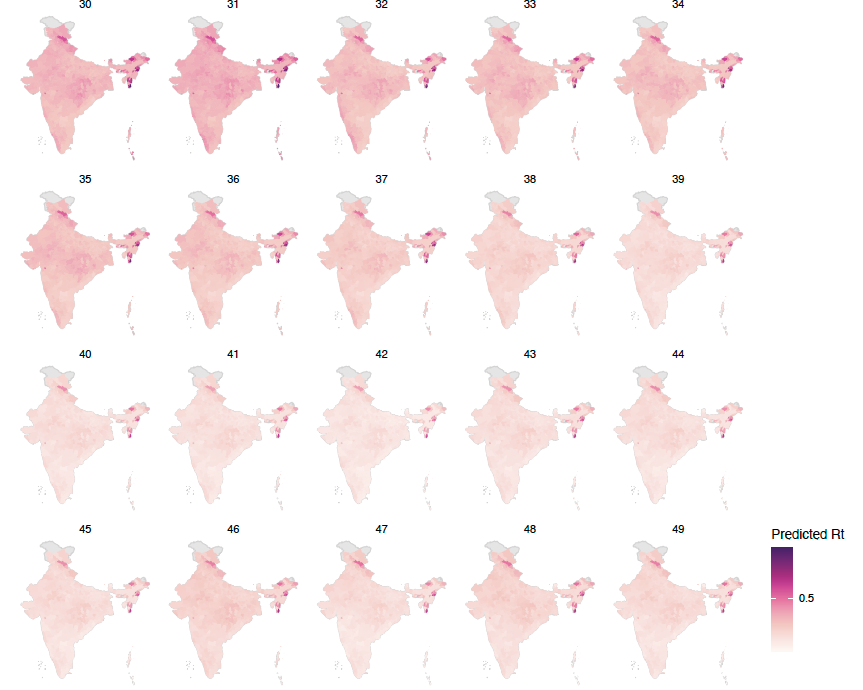


**S38 Fig**. Posterior predictive mean Rt during wave 1 in India, 2020, derived from the best fitting model (model 4.1) at country level using leave-one-week-out cross-validation approach. The weeks in 2020 investigated are numbered in maps. Areas shaded in grey are areas for which no data is available.


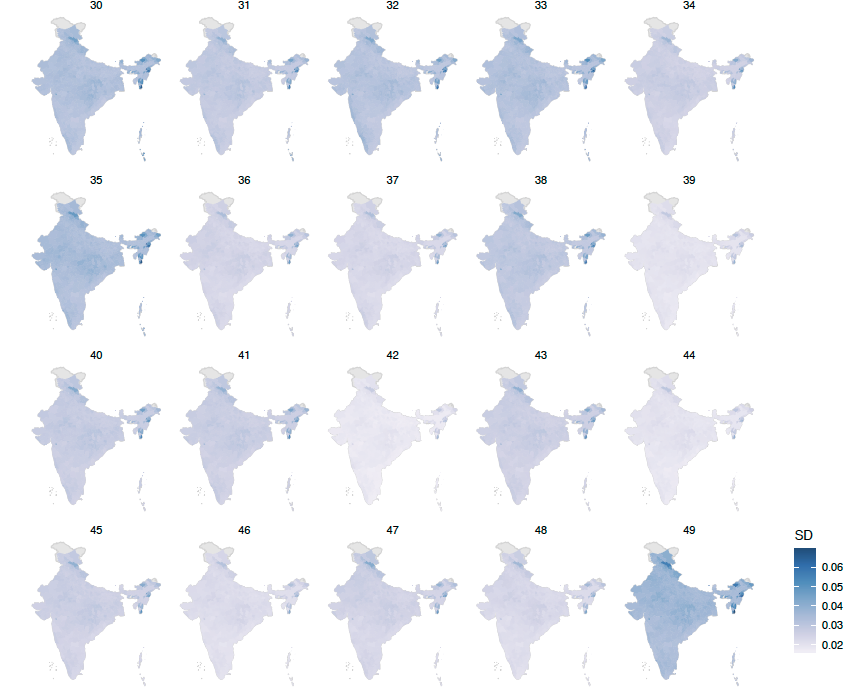


**S39 Fig.** Standard deviation (SD) of posterior predictive Rt during wave 1 in India, 2020, derived from the best fitting model (model 4.1) at country level leave-one-week-out cross-validation approach. Areas shaded in grey are areas for which no data is available.


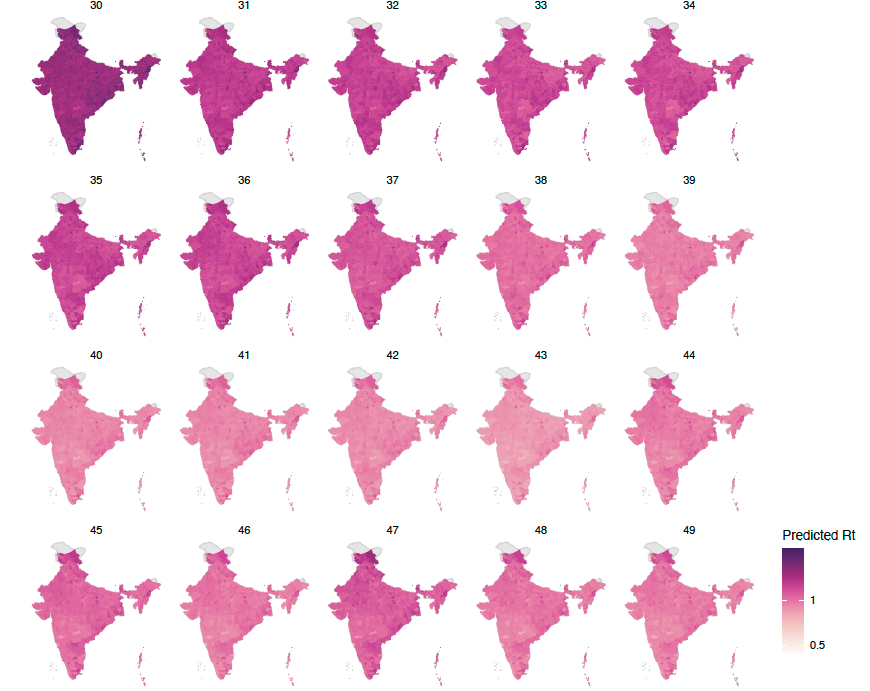


**S40 Fig**. Posterior predictive mean Rt during wave 1 in India, 2020, derived from the best fitting model (model 4.1) at country level using leave-one-district-out cross-validation approach. The weeks in 2020 investigated are numbered in maps. Areas shaded in grey are areas for which no data is available.


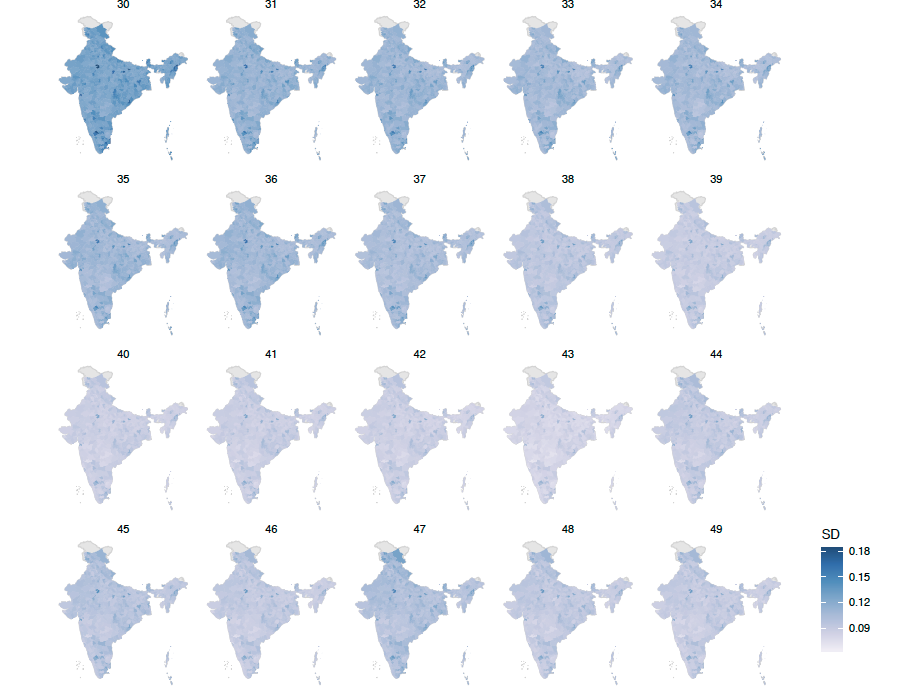


**S41 Fig.** Standard deviation (SD) of posterior predictive Rt during wave 1 in India, 2020, derived from the best fitting model (model 4.1) at country level leave-one-district-out cross-validation approach. Areas shaded in grey are areas for which no data is available.


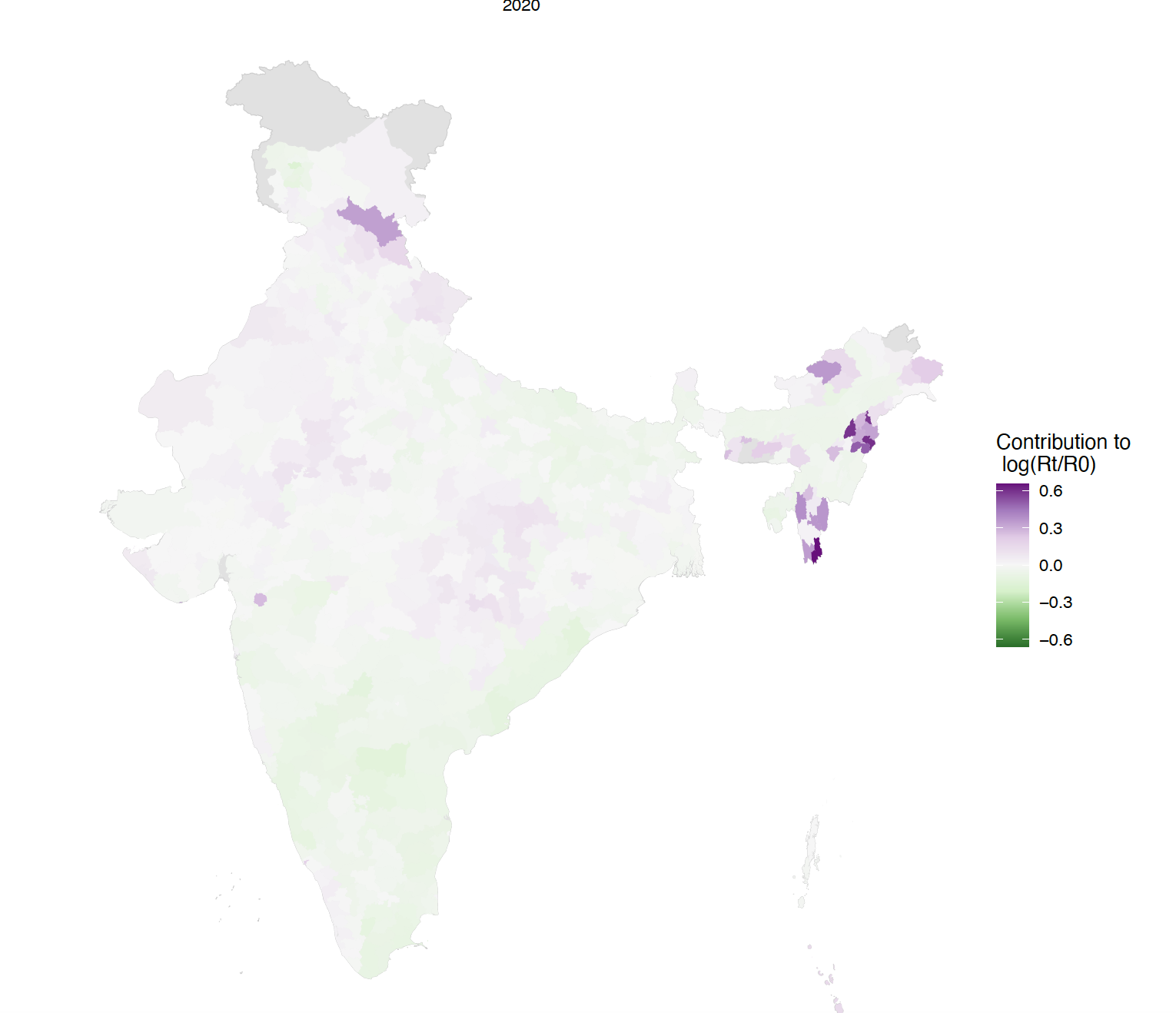


**S42 Fig**. Contribution of spatial random effects to estimates of Rt changes in the base model. Areas shaded in grey are areas for which no data is available.


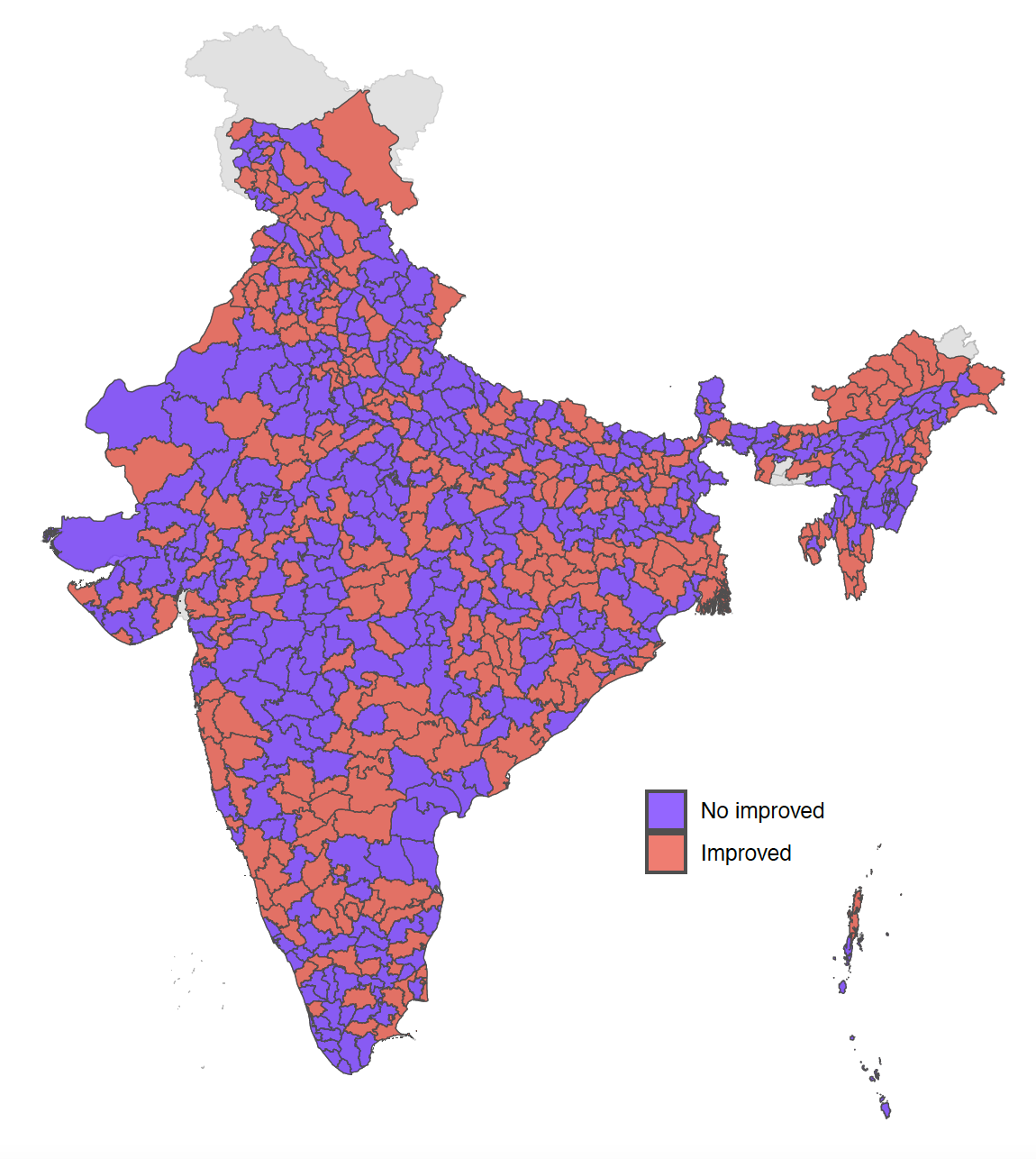
**
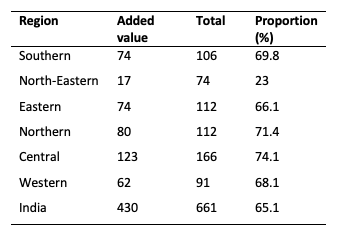
**

**S43 Fig**. Improvement by using the best fitting model across the country, compared to baseline model. Difference between mean absolute error (MAE) for the baseline model (weekly random effects, spatial random effects and population density) and MAE for the best fitting model (model 4.1 with DLNMs). Districts with positive values (pink) suggest that capturing the nonlinear and delayed impacts of mobility, climate information and intervention stringency, improves the model in these areas. Districts with negative values (blue) suggest that mobility, intervention and climate information did not improve the model fit and other unexplained factors might dominate space-time dynamics in these areas. The MAE of the selected model was smaller than the baseline model for 430 of the 661 (65.17%) districts in India, with the results of model performance provided by geo-political regions in the Table. Areas shaded in grey are areas for which no data is available.


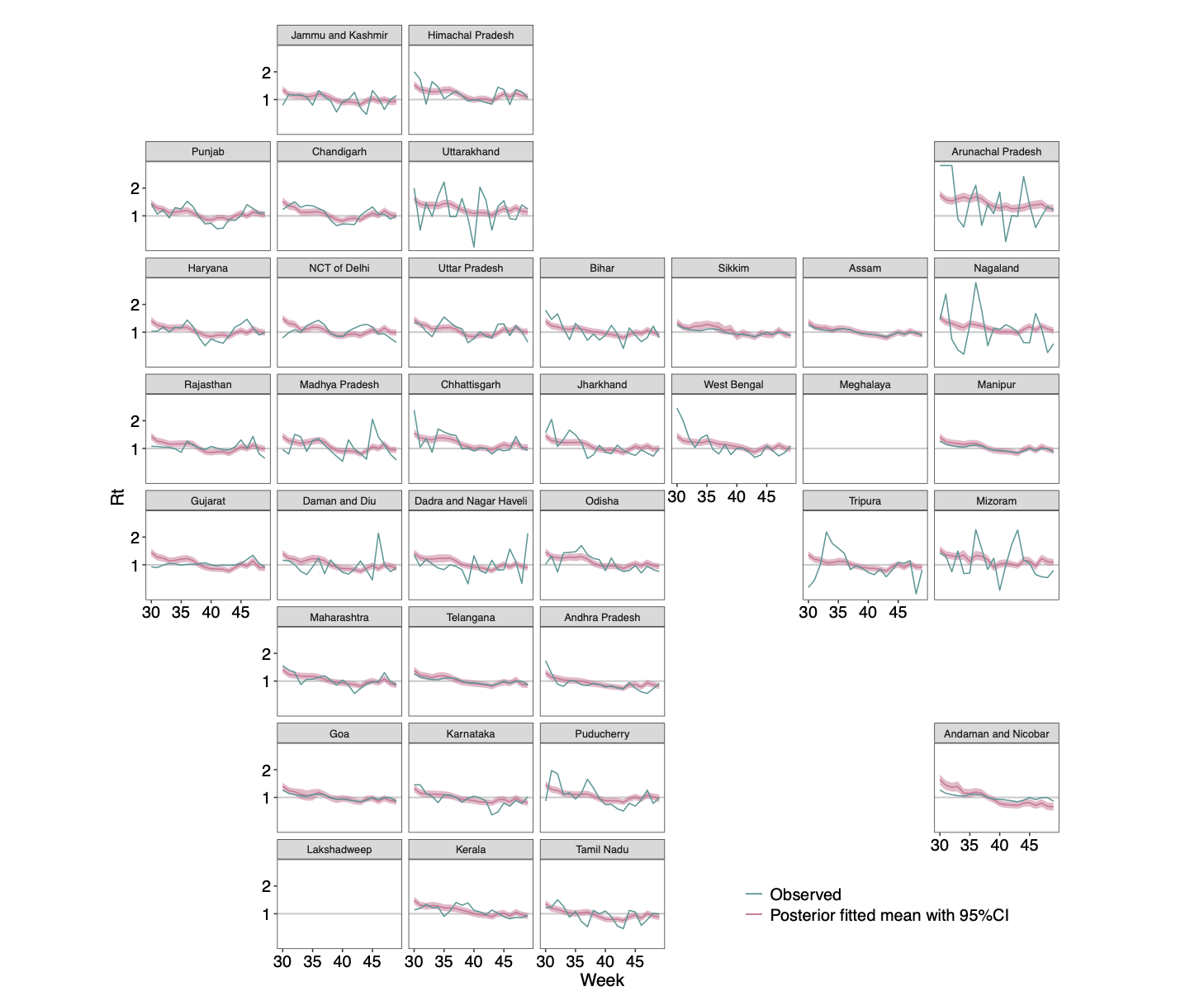


**S44 Fig.** Observed versus posterior fitted Rt in the capital district of each state using the best fitting model (model 4.1 with DLNMs) at country level. Graphs with a log scale at y-axis show the observed Rt derived from reported case data, and corresponding mean and 95% confidence interval (CI, shaded pink area) of fitted Rt, derived from the best fitting model (model 4.1 with DLNMs) at country level. States are ordered by their geographical location.

**S45 Fig.** Observed versus posterior predictive Rt in the capital district of each state, using leave-one-week-out cross-validation approach**.** Graphs with a log scale at y-axis show the observed Rt derived from reported case data, and corresponding posterior predictive mean and 95% prediction interval (CI, shaded pink area), derived from the best fitting model (model 4.1 with DLNMs) at country level. States are ordered by their geographical location.

**
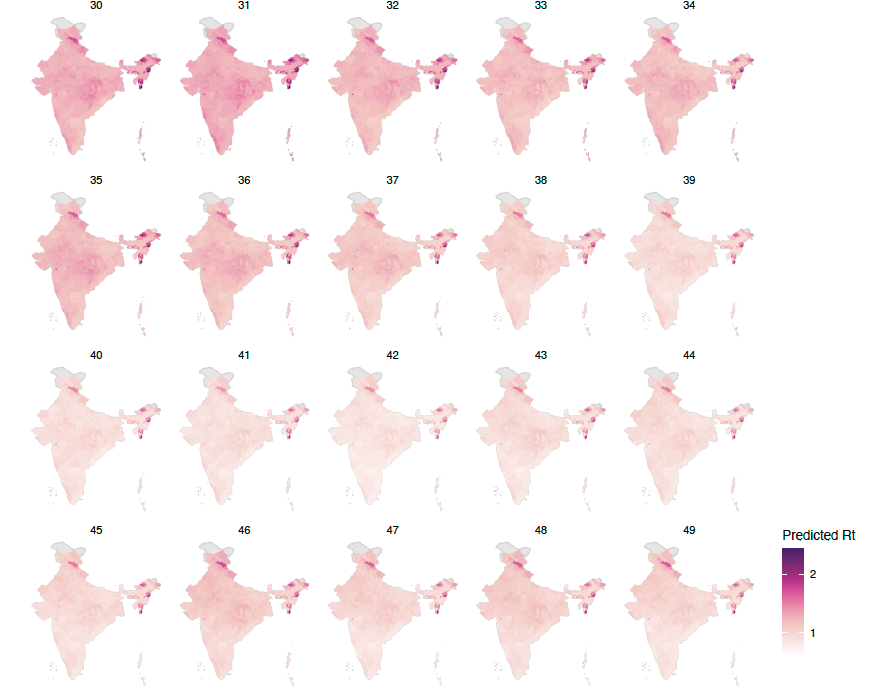
**

**S46 Fig.** Posterior predictive mean Rt during the wave 1 in India, 2020, derived from the best fitting model (model 4.1 without DLNMs) at country level using 2-week lag covariates and leave-one-week-out cross-validation approach. Areas shaded in grey are areas for which no data is available.

**
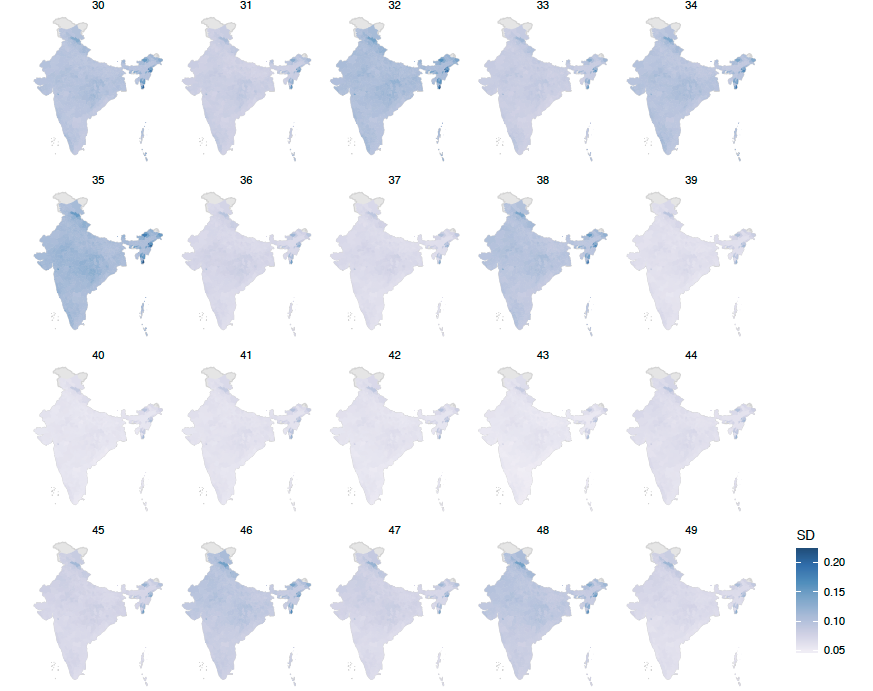
**

**S47 Fig.** Standard deviation (SD) of posterior predictive Rt during wave 1 in India, 2020, derived from the best fitting model (model 4.1 without DLNMs) at country level using 2-week lag covariates and leave-one-week-out cross-validation approach. Areas shaded in grey are areas for which no data is available.

**
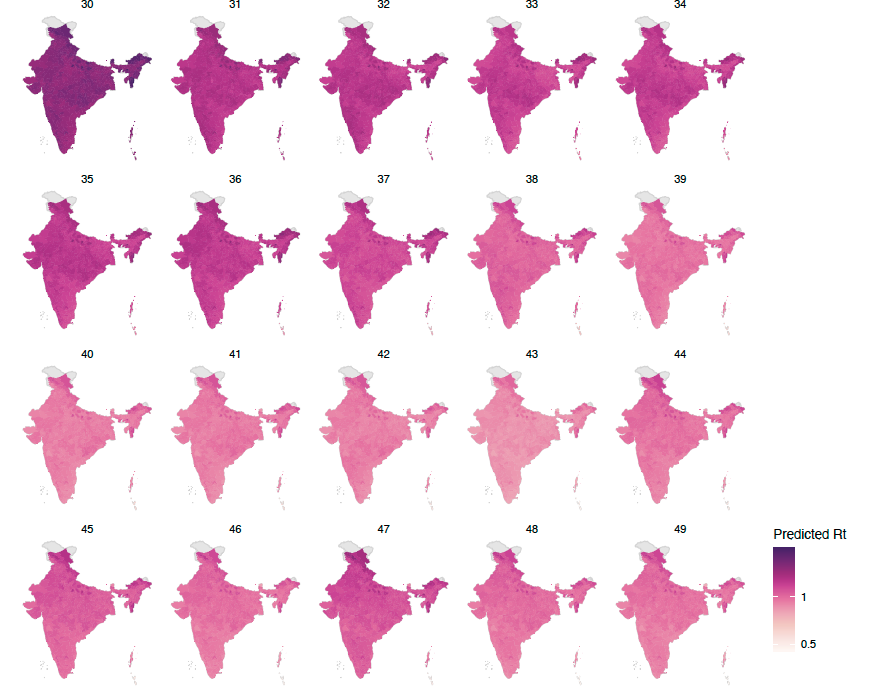
**

**S48 Fig.** Posterior predictive mean Rt during the wave 1 in India, 2020, derived from the best fitting model (model 4.1 without DLNMs) at country level using 2-week lag covariates and leave-one-district-out cross-validation approach. Areas shaded in grey are areas for which no data is available.

**
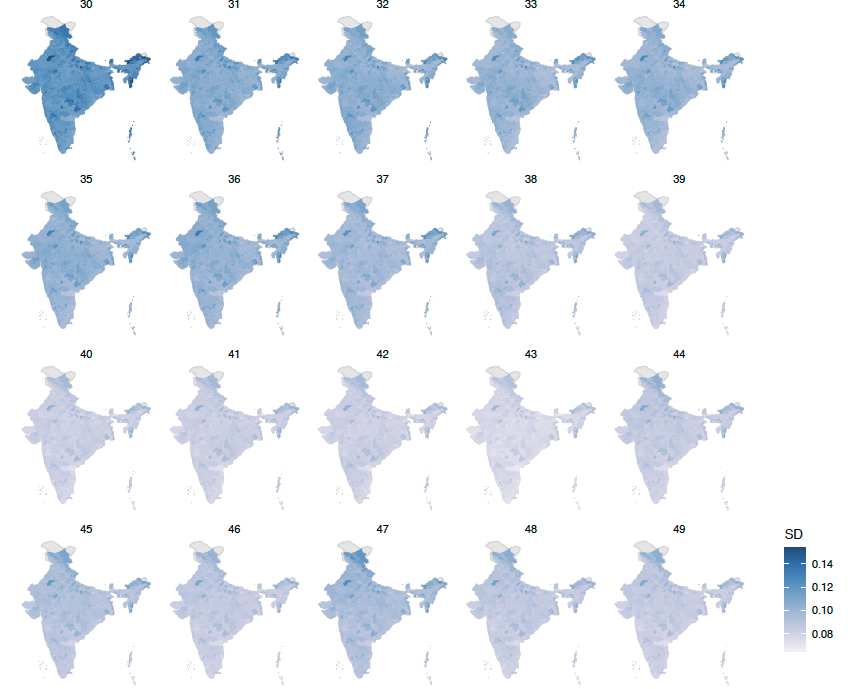
**

**S49 Fig.** Standard deviation (SD) of posterior predictive Rt during wave 1 in India, 2020, derived from the best fitting model (model 4.1 without DLNMs) at country level using 2-week lag covariates and leave-one-district-out cross-validation approach. Areas shaded in grey are areas for which no data is available.
