# Supplementary material for: Comparing lagged impacts of mobility changes and environmental factors on COVID-19 waves in rural and urban India: A Bayesian spatiotemporal modelling study
Source: PLOS Glob Public Health. 2025 Apr 30;5(4):e0003431. doi: 10.1371/journal.pgph.0003431 (PMC12043145; doi:10.1371/journal.pgph.0003431)
Supplement: Attachment — Submitted filename: Response to reviewers.docx [file pgph.0003431.s004.docx]

**Comparing lagged impacts of mobility changes and environmental factors on COVID-19 waves in rural and urban India: a Bayesian spatiotemporal modelling study**

Manuscript number PGPH-D-24-01332

- Additional Editor Comments: Thank you for the revised manuscript. A quick minor but essential suggestion to revise the text before I am able to recommend for further process. I suggest to revise the term 'the instantaneous basic reproduction number (R0) over time' to illustrate the basic reproduction number for each variant as measure of initial transmissibility (without the effects of interventions and no depletion in susceptibility in the population) as 'the variant-specific basic reproduction number (R0) across the waves'. The terms 'instantaneous' and 'over time' indicate the measure is temporal, where R0 has very specific meaning in epidemiology: R0 is a measure of intrinsic transmissibility…Otherwise, it can be termed as the variant-specific initial reproduction number (R_in) across the waves"? Where, R_in is a measure of initial transmissibility (not a temporal measure), still accounts the affects of intervention in the population.

Response: We have changed this in the text with a clear definition of the variant specific R0 (R_in) according to the editors recommendations (lines 259 to 262).

- Reviewer #3: The authors “explored exposure-lag response associations between the relative risk (RR) of increase in COVID-19 transmission, and changes in mobility…”. I think the expression “relative risk” is not appropriate. It can be changed to the ratio of XX.

Response: We have changed this in the text highlighted by the reviewer (line 350) and also at other points in the text where changing the phrasing is appropriate (lines 461, 476, 479 & 480).
