# Supplementary material for: Comparing lagged impacts of mobility changes and environmental factors on COVID-19 waves in rural and urban India: A Bayesian spatiotemporal modelling study
Source: PLOS Glob Public Health. 2025 Apr 30;5(4):e0003431. doi: 10.1371/journal.pgph.0003431 (PMC12043145; doi:10.1371/journal.pgph.0003431)
Supplement: Attachment — Submitted filename: Reviewer comments and author repsonse.docx [file pgph.0003431.s003.docx]

PLOS Global Public Health Manuscript Number: PGPH-D-24-01332

**Reviewer comments and author response**

**Reviewer #1**

I am writing to request higher resolution figures for our paper to facilitate a smoother review process. The current resolution makes it difficult for reviewers to thoroughly evaluate the details. I have already informed the editor about this issue, but I have not yet received the necessary documents. Your prompt assistance in providing these higher resolution figures would be greatly appreciated.

**Response:**

To the reviewer, we sincerely apologise for the poor resolution of the uploaded figures. We believe this issue arose with conversion of image formats during the submission process.

We were made aware of this issue by the original editor assigned to our manuscript and we made several efforts to rectify this issue. However, there is no facility for uploading revised documents on the editorial manager system while the manuscript is under peer review. We tried to send new documents to the reviewer via email contact with the journal but unfortunately received no response as to how to do this.

We have now uploaded new high resolution images and we sincerely apologise for the delay in amending this issue. We have also uploaded our manuscript to a preprint server with high resolution figures that can be viewed here (<https://www.medrxiv.org/content/10.1101/2024.06.12.24308871v1>) if this problem persists.

**Reviewer #2**

Expand the literature review: while the introduction gives a good overview, it could benefit from a broader discussion of existing research. Consider adding more recent studies (from 2022-2023) on the relationships between mobility, climate factors, and COVID-19, especially those using similar spatiotemporal modeling approaches in other regions.

**Response:** Thank you for this comment. We have broadened our literature review and expanded the introduction (lines 82 to 100) with research published between 2020 and 2024.

Clarify the research gap: while the gap in previous studies is mentioned, more emphasis on why this study is critical in the global pandemic context, not just in India, would strengthen the rationale for the research.
**Response:** Thank you for this comment. We have expanded the introduction and edited the text to clarify the gap (lines 74 to 105).

The technical aspects of the Bayesian spatiotemporal hierarchical models and INLA are not well explained and should be stated clearly. How the drivers are included in the spatiotemporal hierarchical models and INLA. Briefly explain why certain variables (such as temperature and precipitation) were selected and how they contribute to the model.

**Response:** Thank you for drawing our attention to this. We have included an overview of Bayesian spatiotemporal frameworks and the Integration Nested Laplace Approximation approach in the methods section (lines 304 to 313).

We have also included information about how the models were structured and covariates were included in the models (lines 317 to 327).

Additional information has been included in the introduction about previous published research exploring contribution of climate drivers to COVID-19 transmission as justification for investigating climate impacts of COVID-19 on daily covid cases in India (lines 87 to 99). We have also included additional information about variable selection prior to inclusion in Bayesian spatiotemporal models in the methods section (lines 321 to 324).

Justify time lags: more explanation is needed about the rationale for choosing specific time lags (e.g., 0-3 weeks). Why were these particular lag intervals chosen? This can help reinforce the validity of the modelling approach.

**Response:** Thank you for this comment. The lag times (0-3 weeks) were chosen to account for any delays in reporting and the estimated delay in impact of interventions on transmission. This lag is consistent with previously published research exploring the lagged impact of meteorological conditions on COVID-19 transmission across eight different countries in Europe, Africa, Asia and South America doi: [10.1186/s12967-022-03371-1](https://doi.org/10.1186/s12967-022-03371-1).

We have included further explanation as to choice of time lags in the main manuscript and referenced previous research using the same time lags in our manuscript.

Streamline the presentation: some parts of the results section are a little bit repetitive. Streamlining the discussion of the findings and emphasizing the most significant and novel results (especially regarding the role of mobility and intervention stringency) will improve readability.

**Response:** Thank you for this comment, we have edited the results and discussion sections based on this comment.

Explain spatial random effects more clearly: the explanation of spatial random effects is a bit technical and could be clearer. A more intuitive description of what these effects represent (e.g., unmeasured regional differences) would help the reader understand their importance.

**Response:** Thank you for drawing our attention to this. We have rewritten this paragraph to improve clarity for the reader (lines 329 to 344).

**Reviewer #3**

I think the following sentence may be inappropriate: “However, no previous research has compared mobility patterns, or inter-district movement across both pandemic waves, relative to pre-pandemic mobility levels, and associated impact on COVID-19 transmission.”, since there have been studies comparing mobility patterns.

**Response:** Thank you, we have clarified this sentence to emphasise the novelty of our approach using fine spatial scale aggregated mobility data, as opposed to Google mobility report data used by previous published research identified by the authors (<https://doi.org/10.1016/j.sste.2021.100442>; <https://doi.org/10.1111/1745-5871.12502>). We have also clarified that in this context the research gap refers specifically to India (lines 74 to 77).

The authors mentioned that they used “pre-pandemic mean baseline levels of mobility for the first eight weeks of 2020”. Sensitivity analysis can be conducted to change “eight weeks” to others.
**Response:** This is an interesting point and one which we have investigated. We have explored mobility changes compared to baseline at two week intervals from 4 to 12 weeks (Figure 1) and percentage change compared to 8 week baseline values at 4, 6 , 10 and 12 weeks (Figure 2). These analyses show no discernible difference in percentage mobility changes at different time intervals and so we have retained the eight-week baseline time period used in our models.

**Figure 1. Mobility changes relative to different baseline lengths in India**

**Figure 2. Percentage changes compared to 8-week baseline values by district in India**

The authors mentioned that “Public holidays, which included the date of public holiday and one day before and after, were assigned a value of 1”. Sensitivity analysis can be conducted to change “the date of public holiday and one day before and after” to others.

**Response:** Thank you for the comment. In our models we have accounted for weekly temporality in the data, and we expect changes in mobility to be observed one day before and one day after a public holiday. We therefore conclude that including a binary variable for public holidays with 1 denoting the holiday day and 0 denoting days not deemed public holidays to be more effective at capturing the effect of any mobility change associated with public holidays in our model.

I think the expression “the instantaneous basic reproduction number ( 0) over time” may be not appropriate, since 0 does not change over time.

**Response:** Thank you drawing our attention to this. This appears to be a typo in the text. We have double checked this sentence and have clarified it to, ‘we also estimated the instantaneous basic reproduction number ($R_{0})$ over time to capture the intrinsic transmission capability of different variants without interventions.’

We conclude that some text may have been omitted in the conversion of the original Word document to PDF format during the manuscript submission process.

How to determine the parameters of the gamma distribution for △ | _ (i.e., /0.5, 0.5)? The authors can provide references for this or give reasons.

**Response:** A gamma distribution was determined based on the distribution of the observed data and based on the lowest deviance information criterion (DIC) during initial exploratory analyses using Weibull, Gaussian and gamma distributions in our base model. Please see additional information [here](https://github.com/e3cleary/COVID-19-INDIA/blob/main/delta_wave/Rcode/3_run_models.R) (lines 44 & 49).

The authors “explored exposure-lag response associations between the relative risk (RR) of increase in COVID-19 transmission, and changes in mobility…”. I think the expression “relative risk” is not appropriate, since △ is not a binary variable.

**Response:** Thank you for this comment. In this instance the RR is the correct estimate of associations with drivers of COVID-19 transmission as our dependent variable is change in COVID Rt, the effective reproduction number representing the transmission capability of the virus over time, and a continuous variable. Please see variable description below (Figure 3).

**Figure 3. Distribution of Rt during the second pandemic wave in India.**
